# 3β-*O*-Tigloylmelianol from *Guarea kunthiana*: A New Potential Agent to Control *Rhipicephalus (Boophilus) microplus*, a Cattle Tick of Veterinary Significance

**DOI:** 10.3390/molecules20010111

**Published:** 2014-12-23

**Authors:** Carlos Henrique Miguita, Carolina da Silva Barbosa, Lidilhone Hamerski, Ulana Chaves Sarmento, José Nicácio do Nascimento, Walmir Silva Garcez, Fernanda Rodrigues Garcez

**Affiliations:** 1 Laboratório de Pesquisa de Produtos Naturais Bioativos, Instituto de Química, Universidade Federal de Mato Grosso do Sul, 79074-460 Campo Grande, MS, Brazil; E-Mails: miguita_carloshenrique@hotmail.com (C.H.M.); ulanachaves@hotmail.com (U.C.S.); inqui@ufms.br (W.S.G.); 2 Laboratório de Parasitologia Animal, Universidade Estadual de Mato Grosso do Sul, 79200-000 Aquidauana, MS, Brazil; E-Mail: csmangarosa@gmail.com; 3 Instituto de Pesquisas de Produtos Naturais, Universidade Federal do Rio de Janeiro, 21941-902 Rio de Janeiro, RJ, Brazil; E-Mail: hamerski_l@hotmail.com; 4 Laboratório de Insetos Frugíforos, Faculdade de Ciências Biológicas e Ambientais, Universidade Federal da Grande Dourados, 79804-970 Dourados, MS, Brazil; E-Mail: nicacioj.n@gmail.com

**Keywords:** tick control, *Guarea kunthiana*, protolimonoids, *Rhipicephalus (Boophilus) microplus*, biopesticides

## Abstract

Chemical investigation of *Guarea kunthiana* fruits, guided by their effect on the reproductive cycle of engorged females of the cattle tick *Rhipicephalus (Boophilus) microplus*—a major economic problem to the livestock industry worldwide—led to isolation of 3β-*O*-tigloylmelianol, a new protolimonoid, from the bioactive hexane phase obtained by partitioning the crude ethanol extract. An adult immersion test was performed. The compound strongly inhibited egg-laying and hatchability (99.2% effectiveness at a 0.01% concentration). Melianone, isolated from the same phase, yielded unremarkable results in the adult immersion test. From the dichloromethane phase, melianol, melianodiol, meliantriol, and a new protolimonoid, 3β-*O*-tigloylmeliantriol, were isolated, all of which, in the same manner as melianone, exhibited unremarkable results in the test. The structures of new and known compounds were mostly established by 1D- and 2D-NMR analyses and mass spectrometry data. This is the first report on the bioactivity of protolimonoids on the reproductive cycle of engorged females of *R. (B.) microplus*. 3β-*O*-Tigloylmelianol proved a promising candidate for the development of a biocontrol agent against the cattle tick investigated, as an alternative to environmentally hazardous synthetic acaricides.

## 1. Introduction

Midwest Brazil harbors the country’s largest zebu herd, which is susceptible to parasites such as the cattle tick *Rhipicephalus (Boophilus) microplus* Canestrini, vector of the protozoan hemoparasites *Babesia bovis* Starcovici and *Babesia bigemina* Mith & Kilborne and the rickettsia *Anaplasma marginale* Theiler, causative agents of cattle tick fever, a severe disease responsible for productivity losses in several sectors of the livestock industry, affecting particularly *Bos taurus* and its crosses [1,2]. For its significant morbidity and mortality, this tick-borne parasitic infection ranks among the most economically relevant arthropod-transmitted cattle diseases worldwide [3,4]. In Brazil, losses caused by *R. microplus* and the pathogens it transmits reach US$3.24 billion annually [5]. Traditional control of this ectoparasite by continuous, indiscriminate use of synthetic acaricides has had deleterious consequences, including their undesirable environmental persistence, high toxicity to non-target organisms, and contamination of cattle-derived products, in addition to the emergence of resistant tick strains [3,6,7,8,9]. These issues have stimulated the search for new environmentally and toxicologically safe acaricides, increasing the interest in natural products as alternative agents to the synthetic acaricides commonly employed in veterinary pest management [4,9,10,11]. In a previous study on plant extracts as potential sources of natural acaricidal agents, our group investigated ethanol extracts from a large number of species occurring in the Cerrado and Pantanal ecosystems of Midwest Brazil, for their effects on the reproductive cycle of *R. (B.) microplus* [10]. Of the crude extracts evaluated, that obtained from the fruits of *Guarea kunthiana*, a meliaceous species growing in the Cerrado, exhibited 99.1% of product effectiveness at a 0.2% concentration. In the present study, this extract was submitted to a bioactivity-guided chemical investigation using the adult immersion test, in order to isolate and characterize the compounds responsible for the inhibitory effects on oviposition by engorged cattle ticks.

## 2. Results and Discussion

**Figure 1:**
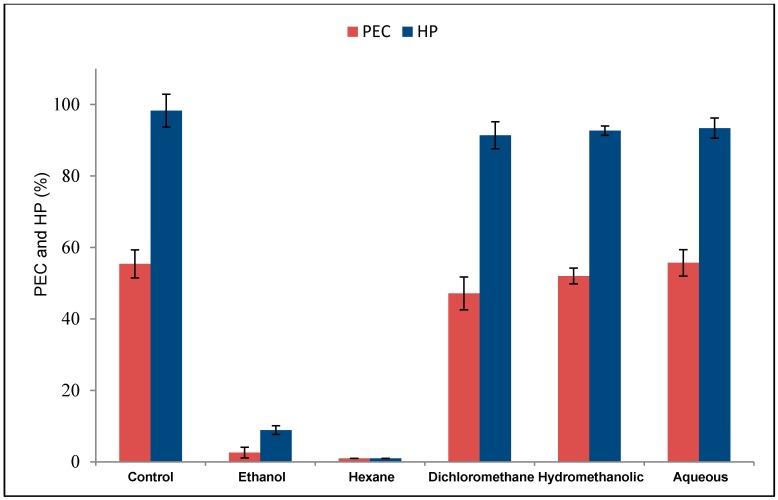
Effects (mean ± SD) of ethanol extract and phases obtained from its partition (each at 0.2%) *vs.* effect of control on % of egg conversion (PEC) and hatching % (HP) for engorged females of *R. (B.) microplus*. ANOVA; Tukey *post-hoc* comparison.

After partitioning of the bioactive ethanol extract of *G. kunthiana* fruits, the resulting hexane, dichloromethane, hydromethanolic, and aqueous phases were evaluated *in vitro* for their efficacy against engorged females by assessing the inhibitory effects on oviposition and hatchability. The hexane phase displayed the highest efficacy, inhibiting egg laying and hatching by 100% (100% product effectiveness at a 0.2% concentration), while unremarkable results were obtained from the other phases at the same concentration, as revealed by their rates of egg-laying and hatchability inhibition, compared with those of controls (Table 1 and Figure 1).

**Table 1:** Means ± SD of % of egg conversion (PEC), hatching % (HP), and product effectiveness (PE) for engorged females of *R. microplus* treated with the ethanol (EtOH) extract of *G. kunthiana* fruits and phases obtained from its partition—namely, aqueous (H_2_O), hexane, dichloromethane (CH_2_Cl_2_) and hydromethanolic (MeOH-H_2_O) phases at different concentrations.

Extract/Phases	Concentration (%)								
	0.20			0.10			0.05		
	PEC	HP	PE	PEC	HP	PE	PEC	HP	PE
EtOH	2.6 ± 1.5 *	8.9 ± 1.2 *	99.5 ± 0.3 *	18.7 ± 6.4 *	23.3 ± 8.8 *	90.2 ± 6.8 *	33.2 ± 3.4 *	16.2 ± 7.7 *	89.0 ± 4.1 *
H_2_O	55.7 ± 3.7	93.4 ± 2.8	1.5 ± 3.5	53.7 ± 6.7	97.8 ± 2.3	4.0 ± 1.1	54.8 ± 1.2	95.6 ± 0.1	0.6 ± 1.8
Hexane	0.0 ± 0.0 *	0.0 ± 0.0 *	100.0 ± 0.0*	4.1 ± 7.1 *	4.8 ± 8.2 *	98.8 ± 2.0 *	5.6 ± 5.2 *	9.7 ± 8.7 *	98.3 ± 1.7 *
CH_2_Cl_2_	47.1 ± 4.6	91.4 ± 3.8	9.6 ± 3.3	48.6 ± 2.2	94.1 ± 6.3	2.5 ± 1.1	48.3 ± 2.2	98.3 ± 2.8	4.1 ± 1.9
MeOH-H_2_O	52.0 ± 2.2	92.7 ± 1.3	16.5 ± 4.1	48.9 ± 3.5	97.2 ± 2.5	17.6 ± 7.9	53.3 ± 1.0	96.9 ± 2.7	10.7 ± 1.0

ANOVA; Tukey *post-hoc* comparison; * *p* < 0.05; Control: PEC = 55.4% ± 3.9%; HP = 98.3% ± 4.6%.

**Figure 2:**
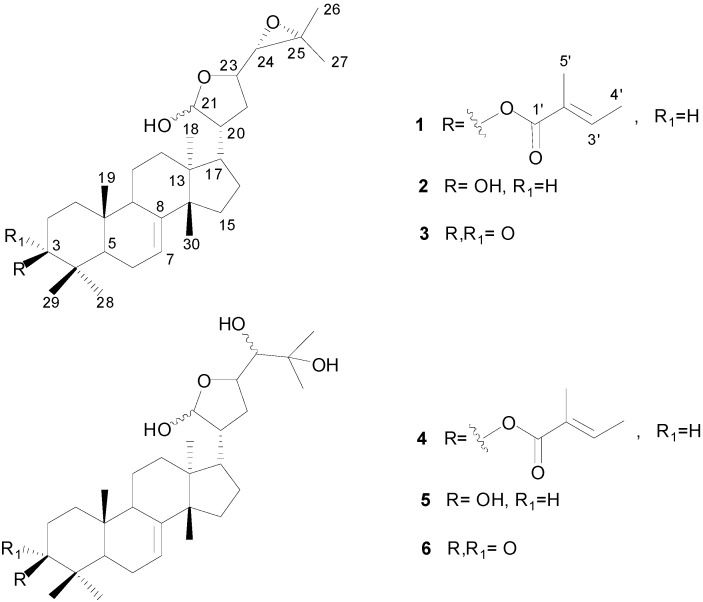
Structures of protolimonoids **1**–**6** isolated from *G. kunthiana*.

Applying a bioassay-directed column chromatography fractionation approach to the highly efficacious hexane phase, only two of the eight fractions obtained proved active (fractions D and E), as shown by their effect on egg production and hatching (98.9% and 99.9% product effectiveness, respectively, at a 0.1% concentration) (Table 2). Even at the lowest concentration tested (0.025%), fractions D and E still showed significant inhibitory effects on egg laying and hatchability (95.9% and 99.0% of product effectiveness, respectively) (Table 2). Further fractionation of these bioactive fractions yielded compounds **1** and **3** (Figure 2).

**Table 2:** Means ± SD of % of egg conversion (PEC), hatching % (HP), and product effectiveness (PE) for engorged females of *R. microplus* treated with different concentrations of fractions A–H obtained from the bioactive hexane phase.

Fractions	Concentration (%)								
	0.1			0.05			0.025		
	PEC	HP	PE	PEC	HP	PE	PEC	HP	PE
A	45.6 ± 3.4	90.3 ± 7.1	9.1 ± 4.0	44.4 ± 2.0	96.5 ± 3.0	5.8 ± 2.0	43.1 ± 2.6	99.2 ± 1.4	5.9 ± 1.9
B	40.8 ± 2.9	91.5 ± 3.5	25.8 ± 8.2	47.4 ± 3.1	92.5 ± 11.0	13.7 ± 2.1	44.8 ± 2.0	99.2 ± 1.4	1.5 ± 0.1
C	44.2 ± 1.4	93.8 ± 4.4	15.2 ± 6.4	45.0 ± 3.3	97.5 ± 2.5	10.2 ± 3.6	44.4 ± 2.4	99.2 ± 1.4	10.5 ± 6.0
D	0.3 ± 0.2 *	17.0 ± 2.9 *	98.9 ± 0.1 *	4.8 ± 2.4 *	19.8 ± 5.4 *	97.7 ± 1.3 *	10.3 ± 5.4 *	20.8 ± 7.2 *	95.9 ± 3.1 *
E	0.1 ± 0.1 *	21.6 ± 1.0 *	99.9 ± 0.0 *	2.6 ± 0.6 *	20.6 ± 4.2 *	98.9 ± 0.2 *	4.5 ± 1.2 *	10.9 ± 1.7 *	99.0 ± 0.1 *
F	45.9 ± 2.2	88.0 ± 7.5	11.7 ± 5.9	46.3 ± 4.2	96.6 ± 3.0	2.0 ± 2.1	46.7 ± 3.4	96.3 ± 3.4	1.4 ± 1.1
G	41.0 ± 2.5	93.3 ± 11.6	19.0 ± 3.6	47.5 ± 1.6	88.8 ± 6.2	11.0 ± 4.8	43.3 ± 1.1	98.3 ± 1.4	10.3 ± 0.9
H	46.3 ± 5.8	95.3 ± 4.3	11.6 ± 3.0	50.7 ± 6.7	86.1 ± 11.3	12.8 ± 1.5	48.1 ± 2.2	98.6 ± 2.5	5.0 ± 1.5

ANOVA; Tukey *post-hoc* comparison; * *p* < 0.05; Control: PEC = 54.4% ± 2.8%; HP = 97.1% ± 4.6%.

The molecular formula of **1** was established as C_35_H_54_O_5_, as deduced from its HRESIMS (*m/z* 577.3865 [M+Na]^+^). Analysis of the ^1^H- and ^13^C-NMR spectra of **1** (Table 3) revealed their high similarity to those of melianol (**2**), a known meliaceous protolimonoid also obtained in the present study, the structure of which has been well established [12].

**Table 3:** ^1^H- (300.13 MHz) and ^13^C- (75.47 MHz) NMR data (CDCl_3_, TMS δ = 0) for 3β-*O*-tigloylmelianol (**1**) and 3β-*O*-tigloylmeliantriol (**4**).

Position	1		4	
	δ_C_	δ_H_, mult. (*J* in Hz)	δ_C_	δ_H_, mult. (*J* in Hz)
1	36.7	1.18–1.24, m	36.8	1.17–1.26, m
		1.62–1.70, m		1.59–1.69, m
2	23.8	1.87–2.01, m	23.8	1.91–2.01, m
		2.03–2.16, m		2.05–2.17, m
3	81.0	4.52, dd (10.9, 3.8)	81.0	4.52, dd (10.8, 4.1)
4	38.1		38.1	
5	50.8	1.41, dd (11.7, 5.5)	50.8/50.9	1.40, dd (11.5, 5.7)
6	24.1	1.52, dd (11.7, 5.4)	24.1	1.61–1.71, m
		1.67, td (11.7, 2.6)		
7	117.9/118.0	5.23, brd (2.6)	117.8/117.9	5.25, brd (4.6)
8	145.5		145.5/145.6	
9	48.7/49.6	2.22, t (7.6)/2.23, t (2.6)	48.7/48.9	2.22, t (7.6)/2.23, t (7.6)
10	34.9		34.9	
11	17.5	1.48, dt (7.6, 2.5)	17.5	1.44–1.54, m
12	35.2	1.36, dt (10.6, 2.5)	31.5	1.45–1.55, m
		2.04–2.16, m		1.65, dt (12.2, 3.4)
13	43.6/43.8		43.5/43.6	
14	50.4		50.7/51.0	
15	34.2	1.42–1.60, m	34.2	1.41–1.57, m
16	27.4	1.23–1.37, m	27.2	1.80–1.90, m
		1.78–1.94, m		
17	45.2/47.1	1.98-2.10, m/1.91-2.06, m	45.2	1.96–2.03, m
18	22.5/23.2	0.83, s	23.2/22.4	0.81, s
19	13.1	0.75, s	13.1	0.75, s
20	31.7/33.8	1.43–1.57, m	46.4	1.88–1.98, m
		1.60–1.79, m		
21	97.8/ 101.8	5.30, d (2.5)/5.34, d (2.6)	96.8/102.2	5.23, brs/5.31, brs
22	31.3/31.5	1.38–1.48, m	30.3	1.84–1.91, m
		1.94–2.02, m		
23	77.0/78.4	3.84, dt (9.5, 75)/	77.0/78.6	4.32–4.35, m/
		3.90, ddd (10.6, 7.7, 5.1)		4.49–4.54, m
24	65.4/67.8	2.68, d (7.5)/2.83, d (7.5)	75.0/75.8	3.11, brs/3.19, brs
25	57.2/57.9		73.1/73.6	
26	19.2/19.4	1.28, s	26.7	1.22, s
27	24.9/25.0	1.28, s	26.7	1.25, s
28	27.6	0.83, s	27.6	0.83, s
29	16.0	0.95, s	16.0	0.95, s
30	27.3	0.95, s	27.3	0.96, s
1'	167.9		168.0	
2'	129.2		129.1	
3'	136.7	6.81, brq (7.1)	136.7	6.82, qq (6.8,1.3)
4'	14.3	1.76, d (7.1)	14.3	1.76, d (6.8)
5'	12.1	1.80, brs	12.0	1.81, d (1.3)

**Figure 3:**
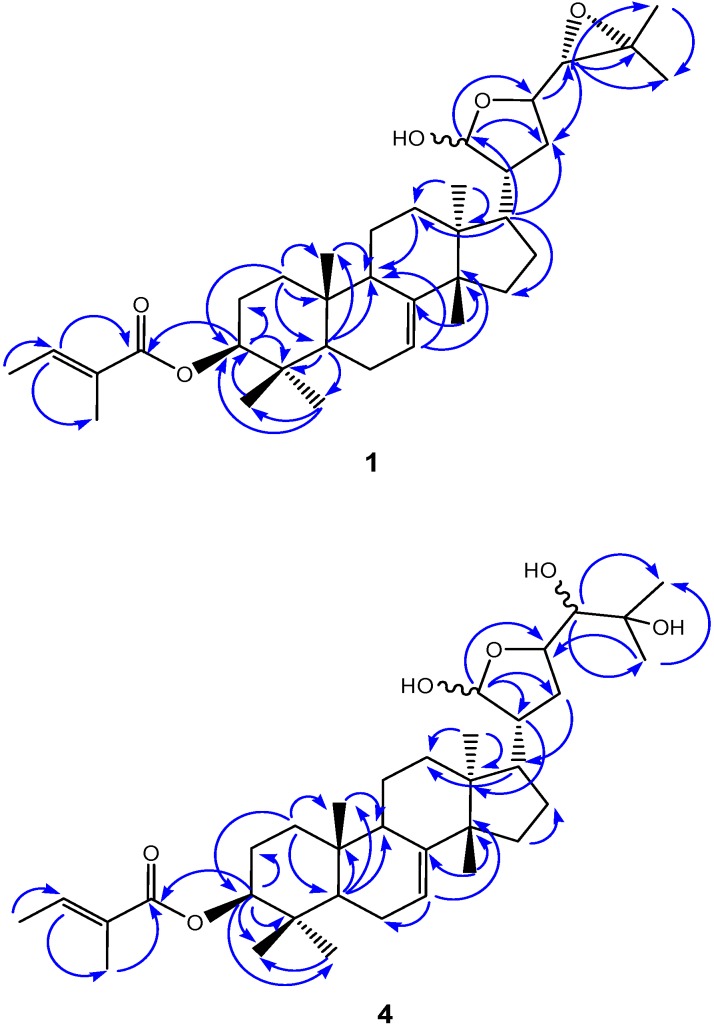
Key HMBC correlations for **1** and **4**.

This reveals that they have identical ring arrangements and side chain at C-17 as well. Two epimeric forms at C-24 could account for some duplicated signals observed in the ^1^H- and ^13^C-NMR spectra of **1**, **2**, and other protolimonoids bearing a hemiacetal carbon at the side chain. Compounds **1** and **2** were shown, however, to differ only in the nature of the substituent attached at C-3: a tigloyloxy residue in **1**, as shown by the presence of characteristic signals in the ^1^H-NMR spectrum at δ 1.76 (d, *J* = 7.1 Hz) and 1.80 (brs), ascribed to vinylic methyls, and δ 6.81 (brq, *J* = 7.1 Hz), attributed to a β-carbonyl olefinic hydrogen, and by carbon resonances at δ 129.2, 136.7, 12.1, 14.3, and 167.9, corresponding to carbons in the trisubstituted double bond, vinylic methyls, and a conjugated ester carbonyl, respectively [13]. The significantly downfield shifted one-proton double of doublets at δ 4.52 (*J* = 10.9, 3.8 Hz), which showed cross-peak correlation with the carbon resonance at δ 81.0 in the HSQC spectrum, was then ascribed to 3-H. HMBC correlations from methyl-28 (δ_H_ 0.83) and methyl-29 (δ_H_ 0.95) to C-3 (δ_C_ 81.0) further supported these assignments, while a long-range connectivity discernible between 3-H and the carbonyl carbon of the tigloyloxy moiety at δ 167.9 confirmed the location of this group at C-3 (Figure 3). The presence of the tigloyloxy residue in **1** also accounted for deshielding of C-3 from **1** (δ 81.0) to **2** (δ 78.9). The upfield shifted resonance of C-2 in **1** (δ 23.8), compared with that of **2** (δ 27.4), provided further support for this assignment. The stereochemistry of 3-H was deduced from its vicinal coupling constants. The observed ^3^*J*_3,2_ values (10.9 and 3.8 Hz) indicated 3-H to be axially oriented and revealed the resulting β-orientation of the tigloyloxy group. Therefore, the structure of protolimonoid **1**, which is being described for the first time, was determined as 3β-*O*-tigloylmelianol (Figure 2). Further evidence for structure **1** was provided by additional two- and three-bond correlations detectable in the HMBC spectrum (Figure 3).

The ^1^H- and ^13^C-NMR spectra of **1** also exhibited a striking resemblance to those described earlier for 3α-tigloylmelianol, an epimeric protolimonoid isolated from *Melia azedarach* (Meliaceae) [14]. In 3α-tigloylmelianol, 3-H was observed as a broad singlet at δ 4.68, which established its equatorial position.

The β-equatorial orientation of the C-3 tigloyloxy group in **1** was responsible for the significant differences seen in the chemical shifts of the A-ring carbons, compared with those of 3α-tigloylmelianol from *M. azedarach*. The β-orientation of this substituent in **1** was in accordance with the γ-effect observed for C-29 (δ 16.0), while the C-29 carbon resonance in 3α-tigloylmelianol was reported to be δ 21.4. Likewise, the equatorial orientation of the tigloyloxy group in **1** had a deshielding effect on C-3 (δ 81.0), which was observed at δ 78.3 in the corresponding epimer. On the other hand, its axial nature in 3α-tigloylmelianol accounted for its γ-effect on C-1 (δ 32.1) and C-5 (δ 46.1) resonances, which were shielded by 4.6 and 4.7 ppm, respectively, when compared with those of **1**.

The NMR data of **3** closely resembled those of melianol (**2**), except for the presence of a C-3 ketone carbonyl in the former, instead of a hydroxyl. This inference was supported by the absence of the double doublet at δ_H_ 3.22 (*J* = 10.1, 3.1), the absence of the carbon resonance at δ 78.9 in the spectra of **3**, both of which were assigned to 3-H/C-3 in **2**, and the presence of a characteristic signal assignable to a carbonyl carbon at δ_C_ 216.9. Furthermore, downfield shifts of the C-2 and C-4 resonances (δ 34.8 and 47.8, respectively) were observed in **3**, compared with those of **2** (δ 27.4 and 38.9, respectively), thus confirming the location of the carbonyl functionality at C-3. These data allowed identification of **3** as melianone (Figure 2), a known protolimonoid [14] that had already been isolated from *Guarea* species—e.g., *G. guidona* (also known as *G. guidonia*) [15] and *G. grandiflora* [16].

**Figure 4:**
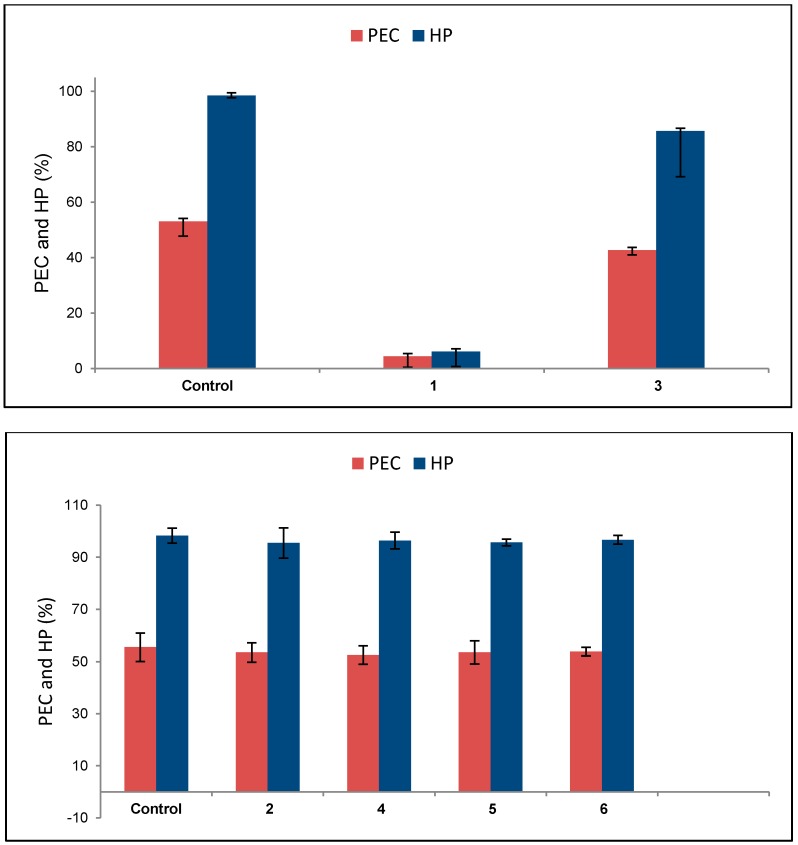
Effects (mean ± SD) of compounds **1** and **3** (each at 0.01%) and compounds **2**, **4**–**6** (each at 0.015%) *vs.* effect of controls on % of egg conversion (PEC) and hatching % (HP) for engorged females of *R. (B.) microplus*. ANOVA; Tukey *post-hoc* comparison.

3β-*O*-Tigloylmelianol (**1**) and melianone (**3**), isolated from the most bioactive fractions obtained from the hexane phase, were then evaluated for their effects on the reproductive cycle of engorged cattle tick females. As depicted in Table 4 and Figure 4, only compound **1** proved highly efficacious, showing noteworthy inhibitory effects on egg production and hatchability, with 99.2% of product effectiveness at a concentration of 0.01%, while melianone (**3**) showed unremarkable results in this test.

Although the dichloromethane phase was devoid of any significant activity in the adult immersion test, its chemical composition was also investigated, leading to the isolation of compounds **2** and **4**–**10** (Figure 2). Compounds **2**, **5**, and **6** proved to have previously known structures and were identified as the protolimonoids melianol [12], meliantriol [17], and melianodiol [18], respectively. This is the first reported occurrence of **2** and **5** in the *Guarea* genus. Compounds **7**–**9**, which were characterized as the mexicanolide-type limonoids humilinolide E, methyl 2-hydroxy-3β-tigloyloxy-1-oxomeliac-8(30)-enate, and swietenine acetate, respectively, and compound **10**, characterized as the andirobin-type limonoid methyl angolensate, had already been obtained in a previous investigation of *G. kunthiana* fruits [19].

Comparison of the NMR spectra of **4** (Table 3) with those of meliantriol (**5**) revealed their close similarity, with identical A, B, C, and D constituent rings, except for the presence of signals attributed to a tigloyloxy residue at C-3 in **4**, as already observed in the spectra of **1**, therefore indicating that the C-3 hydroxyl in **5** was replaced by a tigloyloxy residue in **4**. This assumption was in accordance with the molecular formula C_35_H_56_O_6_, as determined by the (M+Na)^+^ ion peak at *m/z* 595.3971 in the high resolution ESIMS. This proposal was also in agreement with the downfield-shifted double doublet at δ_H_ 4.52 (*J* = 10.8, 4.1 Hz) attributed to 3-H, while the coupling constant value of 10.8 Hz supported its axial orientation, as in compound **1**, and, accordingly, the equatorial position of the tigloyloxy moiety. In addition, deshielding of C-3 (δ 81.0) and shielding of C-2 (δ 23.8) and C-4 (δ 38.1) in **4**, when compared with their corresponding chemical shifts observed for meliantriol **5** (δ_C_ 79.2, 27.6, and 39.0, respectively), corroborated the tigloyloxy moiety location at the C-3 position. Further information provided by the HSQC spectrum, as well as long-range correlations existing in the HMBC spectrum—e.g., from 3-H to the carbon resonances at δ 23.8 (C-2), 38.1 (C-4), 27.6 (C-28), 16.0 (C-29), and 168.0 (the carbonyl carbon of the tigloyloxy residue)—confirmed these assignments (Figure 3). Protolimonoid **4** was thus shown to be 3β-*O*-tigloylmeliantriol (Figure 2), hitherto unreported in the literature.

Although protolimonoids are known to occur in Meliaceae [12,13,14,15,16,17,18], to date only melianone (**3**), melianodiol (**6**), and 21α-acetylmelianone have been described in species of the genus *Guarea*—namely, *G. grandiflora* and *G. guidona* [15,16].

Based on the results obtained for the protolimonoids **1**–**6** in the adult immersion test (Table 4 and Table 5 and Figure 4), both the 24,25-epoxide ring at the side chain at C-17 and the tigloyloxy group at C-3 in the 21,23-epoxy-21-hydroxytirucallane-type skeleton of **1** seem to play a key role in the inhibitory effect on oviposition by engorged cattle ticks, since the opening of the 24,25-epoxide ring in **1**, as found in **4**, led to an expressive reduction in efficacy.

**Table 4:** Means ± SD of % of egg conversion (PEC), hatching % (HP), and product effectiveness (PE) for engorged females of *R. microplus* treated with different concentrations of 3β-*O*-tigloylmelianol (**1**) and melianone (**3**).

Compound	Concentration (%)								
	0.01			0.005			0.0025		
	PEC	HP	PE	PEC	HP	PE	PEC	HP	PE
**1**	4.4 ± 4.0 *	6.1 ± 5.4 *	99.2 ± 0.7 *	24.4 ± 5.3 *	8.1 ± 3.9 *	96.1 ± 0.9 *	27.2 ± 10.1 *	18.7 ± 7.1 *	88.4 ± 8.1 *
**3**	42.7 ± 1.7	85.7 ± 16.5	29.7 ± 15.8	44.0 ± 1.6	92.6 ± 4.4	22.2 ± 2.0	48.8 ± 4.4	92.1 ± 3.1	14.0 ± 1.2

ANOVA; Tukey *post-hoc* comparison; * *p* < 0.05; Control: PEC = 53.1% ± 5.4%; HP = 98.5% ± 0.8%.

**Table 5:** Means ± SD of % of egg conversion (PEC), hatching % (HP), and product effectiveness (PE) for engorged females of *R. microplus* treated with different concentrations of melianol (**2**), 3β-*O*-tigloylmeliantriol (**4**), meliantriol (**5**) and melianodiol (**6**).

Compound	Concentration (%)								
	0.015			0.0075			0.00375		
	PEC	HP	PE	PEC	HP	PE	PEC	HP	PE
**2**	53.5 ± 3.7	95.5 ± 5.8	3.6 ± 1.4	51.4 ± 1.2	97.3 ± 1.8	5.5 ± 0.5	50.0 ± 1.5	100.0 ± 0.0	3.4 ± 2.9
**4**	52.5 ± 3.5	96.4 ± 3.2	12.5 ± 5.2	53.0 ± 1.7	97.7 ± 2.3	10.5 ± 1.1	55.0 ± 1.1	98.5 ± 2.6	6.3 ± 4.2
**5**	53.5 ± 4.4	95.7 ± 1.3	3.0 ± 0.1	52.4 ± 1.5	98.3 ± 2.9	2.4 ± 0.3	53.5 ± 1.0	98.4 ± 1.6	1.17 ± 0.4
**6**	53.8 ± 1.6	96.7 ± 1.7	1.8 ± 0.9	52.5 ± 1.3	99.5 ± 0.8	0.9 ± 0.1	53.2 ± 0.8	98.3 ± 2.9	0.97 ± 0.9

ANOVA; Tukey *post-hoc* comparison; Control: PEC = 55% ± 0.8%; HP = 98.3% ± 2.9%.

Likewise, compounds **2** and **3**, despite bearing the same side chain at C-17 as **1**, are its corresponding C-3 alcohol and ketone derivatives, respectively, and both proved devoid of any significant activity in the adult immersion test at any concentration tested. In this case, the carbonyl functionality at C-3 seems to exert a higher inhibitory effect on oviposition and hatchability than the hydroxyl functionality at the same carbon, since **2** and **3** showed product effectiveness of 3.6% and 29.7%, respectively, at concentrations of 0.015% and 0.01%, respectively (Table 4 and Table 5). Accordingly, **5** and **6**, which exhibited very low product effectiveness (3.0% and 1.8%, respectively, at a 0.015% concentration), are the corresponding 3,24,25-trihydroxy and 3-keto-24,25-dihydroxy derivatives of **1**, respectively.

## 3. Experimental Section

### 3.1. General Procedures

Optical rotations were determined on a Perkin Elmer 341 polarimeter (Waltham, MA, USA). IR spectra were run on a Bomem-Hartmann & Braun FT-IR spectrometer (Quebec, QC, Canada) using KBr pellets. HRESIMS data were acquired on a UltrOTOF-Q instrument (Bruker Daltonics, Bremen, Germany) with electrospray ionization and operating in positive mode. 1D- and 2D ^1^H- and ^13^C-NMR spectroscopic data were recorded at room temperature in CDCl_3_ (Cambridge Isotope Laboratories, Andover, MA, USA) on a Bruker DPX-300 spectrometer (Karlhue, Germany), operating at 300.13 MHz (^1^H)/75.47 MHz (^13^C). Standard pulse sequences were used for homo- and heteronuclear correlation experiments. Chemical shifts are reported in ppm, using TMS as an internal standard (δ = 0 ppm), and coupling constants (*J*) are expressed in Hertz. Column chromatography procedures were performed on silica gel 60 (70–230 or 230–400 mesh, Merck, Darmstadt, Germany), and silica gel 60 RP-18 (230–400 mesh, Merck). Thin layer chromatography analyses were carried out on pre-coated silica gel GF_254_ plates (Merck).

### 3.2. Plant Material

Fruits of *Guarea kunthiana* A. Juss. were collected in Dois Irmãos do Buriti county, Mato Grosso do Sul state, Brazil, in August 2010. The species was identified by Ubirazilda Maria Resende, from the Universidade Federal de Mato Grosso do Sul (UFMS), and a voucher specimen (no. 11217) has been deposited at the CGMS Herbarium at UFMS.

### 3.3. Extraction

Fruits of *G. kunthiana* (2.5 kg) were cut and extracted overnight with 95% EtOH (3 × 12 L) at room temperature. After concentration under reduced pressure, a portion of the crude EtOH extract (40.0 g) was partitioned between *n*-BuOH (650 mL) and H_2_O (650 mL). The *n*-BuOH phase was concentrated under reduced pressure and subsequently partitioned between MeOH–H_2_O (8:2) and hexane and between MeOH–H_2_O (7:3) and CH_2_Cl_2_, to yield the corresponding hexane (7.85 g), CH_2_Cl_2_ (5.0 g), and hydromethanolic (1.2 g) phases.

### 3.4. Bioassay-Guided Isolation of Active Protolimonoid **1** and Inactive Protolimonoids **2**–**6** and Limonoids **7**–**10** from Hexane and CH_2_Cl_2_ Phases

An aliquot of the bioactive hexane phase (6.75 g) was subjected to column chromatography (CC) over silica gel (70–230 mesh) using step gradient elution with hexane, hexane–EtOAc (98:2, 95:5, 90:10, 80:20, 60:40, 40:60), and EtOAc to give eight fractions (A to H). These were tested for their effects on oviposition by engorged females of *R. microplus*. Only fractions D (hexane–EtOAc, 9:1, 1.65 g) and E (hexane–EtOAc, 8:2, 1.30 g) proved bioactive. Fraction D was shown to be mainly composed by fatty acids, triglycerides, and a terpenoid compound, as delineated by its ^1^H-NMR spectrum. An aliquot of this fraction (1.65 g) was then separated by CC on silica gel (230–400 mesh) eluted with hexane–EtOAc (7:3), followed by CC on silica gel (230–400 mesh) eluted with a hexane–EtOAc gradient system (9:1→8:2), to yield protolimonoid **1** (146.4 mg) as the active compound. Fraction E (1.30 g) was shown to contain two main components, which were characterized as protolimonoids based on the TLC profile and ^1^H-NMR spectrum. Rechromatography of this fraction using a silica gel (230–400 mesh) column (hexane–EtOAc, 8:2) furnished the inactive protolimonoid **3** (0.2 g) and further amounts of **1** (0.7 g). A portion of the CH_2_Cl_2_ phase (2.5 g) was chromatographed on a RP-18 silica gel column (40–63 µm) using H_2_O–MeOH (9:1, 8:2, 6:4, 4:6, 2:8, 1:9) and MeOH as eluents to give seven fractions (A to G). Because fraction E (H_2_O–MeOH, 2:8, 0.85 g) was shown to be composed of the previously isolated limonoids **7**–**10** [19], as delineated by NMR spectra and chromatographic analysis followed by comparison with authentic samples, it was not submitted to any further separation. Fraction F (H_2_O-MeOH 1:9, 1.2 g) yielded protolimonoids **2** (42.5 mg), a mixture of **2** and **3** (115.0 mg), **4** (60.3 mg), **5** (84.6 mg), and **6** (150.5 mg), after CC on silica gel (230–400 mesh) eluted with hexane–EtOAc (8:2→4:6). Further amounts of **4** (37.2 mg) were obtained from fraction G (0.33 g, MeOH) after CC on silica gel, using step-gradient elution with CHCl_3_→CHCl_3_:MeOH (8:2).

*3β-O-Tigloylmelianol* (**1**): colorless amorphous powder; ${\left[\text{α}\right]}_{D}^{23}$ +6.22 (c 0.30, MeOH); HRESIMS *m/z* 577.3865 [M+Na]^+^ (calcd. for C_35_H_54_O_5_Na, 577.3871); IR ν_max_ (cm^−1^): 3440, 2950, 1704, 1650, 1269, 1076, 1018, 817, 756, 735. For ^1^H- and ^13^C-NMR data, see Table 3.

*Melianol* (**2**): colorless amorphous powder; HRESIMS *m/*z 495.3433 [M+Na]^+^ (calcd. for C_30_H_48_O_4_Na, 495.3450), *m/z* 455.3508 [M+H-H_2_O]^+^ (calcd. for C_30_H_47_O_3_, 455.3525); ^1^H-NMR (CDCl_3_): δ 1.03–1.15 (1H, m, 1-Ha); 1.59–1.71 (1H, m, 1-Hb); 1.47–1.69 (2H, m, 2-H); 3.22 (1H, dd, *J* = 10.1, 3.1 Hz, 3-H); 1.25–1.32/1.69–1.82 (1H, m, 5-H); 1.86–2.01 (1H, m, 6-Ha); 2.04–2.17 (1H, m, 6-Hb); 5.24 (1H, brs, 7-H); 2.14–2.27 (1H, m, 9-H); 1.40–1.59 (2H, m, 11-H); 1.27–1.40 (1H, m, 12-Ha); 2.05–2.15 (1H, m, 12-Hb); 1.41–1.61 (2H, m, 15-H); 1.22–1.36 (1H, m, 16-Ha); 1.78–1.94 (1H, m, 16-Hb); 1.93–2.04/1.98–2.09 (1H, m, 17-H); 0.82/0.87 (3H, s, 18-H); 0.72 (3H, s, 19-H); 1.63–1.78 (1H, m, 20-H); 5.30/5.35 (1H, brs, 21-H); 1.34–1.47 (1H, m, 22-Ha); 1.90–2.04 (1H, m, 22-Hb); 3.85–3.90 (1H, m, 23-H); 2.69/2.83 (1H, d, *J* = 7.5 Hz, 24-H); 1.28 (3H, s, 26-H); 1.29 (3H, s, 27-H); 0.94 (3H, s, 28-H); 0.83 (3H, s, 29-H); 0.95/0.97 (3H, s, 30-H); ^13^C-NMR (CDCl_3_): δ 37.1 (C-1); 27.4 (C-2); 78.9 (C-3); 38.9 (C-4); 50.3/50.6 (C-5); 23.9 (C-6); 118.0/118.1 (C-7); 145.4/145.5 (C-8); 48.7/49.6 (C-9); 34.9 (C-10); 17.5 (C-11); 35.1 (C-12); 43.5/43.7 (C-13); 50.6/50.9 (C-14); 33.6/34.0 (C-15); 27.2/27.3 (C-16); 45.1/47.2 (C-17); 22.4/23.1 (C-18)*; 13.0 (C-19)*; 31.5 (C-20); 97.5/101.6 (C-21); 31.1/31.3 (C-22); 77.0/78.3 (C-23); 65.5/67.9 (C-24); 57.4/58.0 (C-25); 19.0/19.2 (C-26); 24.7/24.8 (C-27); 27.5 (C-28); 14.7 (C-29); 27.0/27.2 (C-30). Assignments were confirmed by HSQC and HMBC data. *As confirmed by HMBC connectivities of 19-H to C-5, C-9, and C-10, and of 18-H to C-13, C-14, and C-30, and also by HSQC correlations between 19-H (δ 0.72) and C-19 (δ 13.0), and between 18-H (0.82/0.87) and C-18 (δ 22.4/23.1), the previously reported chemical shift values for C-19 (δ 24.1) and C-18 (δ 13.2) [12] should be interchanged.

*Melianone* (**3**): colorless amorphous powder; HRESIMS *m/z* 493.3279 [M+Na]^+^ (calcd. for C_30_H_46_O_4_Na, 493.3294); ^1^H NMR (CDCl_3_): δ 1.34–1.46 (1H, m, 1-Ha); 1.86–1.98 (1H, m, 1-Hb); 2.17 (1H, dt, *J* = 14.4, 2.1 Hz, 2-Ha); 2.70 (1H, dt, *J* = 14.4, 5.1 Hz, 2-Hb); 1.60–1.74 (1H, m, 5-H); 1.98–2.08 (2H, m, 6-H); 5.28 (1H, brs, 7-H); 2.13–2.31 (1H, m, 9-H); 1.45–1.61 (2H, m, 11-H); 1.25–1.35 (1H, m, 12-Ha); 2.02–2.11 (1H, m, 12-Hb); 1.43–1.57 (2H, m, 15-H); 1.22–1.33 (1H, m, 16-Ha); 1.78–1.92 (1H, m, 16-Hb); 1.90–2.00/1.99–2.08 (1H, m, 17-H); 0.77/0.82 (3H, s, 18-H); 0.94 (3H, s, 19-H); 1.34–1.44/1.73–1.82 (1H, m, 20-H); 5.28/5.31 (1H, brs, 21-H); 1.63–1.76 (1H, m, 22-Ha); 1.90–2.01 (1H, m, 22-Hb); 3.76–3.92 (1H, m, 23-H); 2.65/2.83 (1H, d, *J* = 7.4 Hz, 24-H); 1.23 (3H, s, 26-H); 1.22 (3H, s, 27-H); 0.97 (3H, s, 28-H); 1.04 (3H, s, 29-H); 0.95/0.96 (3H, s, 30-H); ^13^C-NMR (CDCl_3_): δ 38.3/38.4 (C-1); 34.8 (C-2); 216.9 (C-3); 47.8 (C-4); 52.2/52.3 (C-5); 24.2 (C-6); 117.9/118.0 (C-7); 145.4/145.5 (C-8); 48.3/49.6 (C-9); 34.9 (C-10); 17.6 (C-11); 35.0 (C-12); 43.4/43.6 (C-13); 50.6/50.9 (C-14); 34.1 (C-15); 27.2/27.3 (C-16); 45.1/46.9 (C-17); 22.4/23.2 (C-18); 12.6 (C-19); 31.1/33.7 (C-20); 97.6/101.6 (C-21); 31.5/31.6 (C-22); 76.8/78.3 (C-23); 65.3/67.7 (C-24); 57.1/57.9 (C-25); 19.1/19.3 (C-26); 24.8/24.9 (C-27); 24.4 (C-28); 21.5 (C-29); 27.1/27.3 (C-30). Assignments were confirmed by HSQC and HMBC data.

*3β-O-Tigloylmeliantriol* (**4**): colorless amorphous powder; ${\left[\text{α}\right]}_{D}^{23}$ +4.24 (c 0.30, MeOH); HRESIMS *m/z* 595.3971 [M+Na]^+^ (calcd. for C_35_H_56_O_6_Na, 595.3977); IR ν_max_ (cm^−1^): 3444, 2950, 1704, 1651, 1269, 1141, 1076, 1018, 975, 820, 757, 734. ^1^H- and ^13^C-NMR data, see Table 3.

*Meliantriol* (**5**): colorless amorphous powder; HRESIMS *m/z* 513.3539 [M+Na]^+^ (calcd. for C_30_H_50_O_5_Na, 513.3556); ^1^H-NMR (CDCl_3_): δ 3.22 (1H, dd, *J* = 10.3, 2.5 Hz, 3-H); 5.21 (1H, brs, 7-H); 0.81 (3H, s, 18-H); 0.72 (3H, s, 19-H); 5.24 (1H, brs, 21-H); 4.33–4.38/4.45-4.47 (1H, m, 23-H); 3.14/3.19 (1H, brs, 24-H); 1.23 (3H, s, 26-H); 1.26 (3H, s, 27-H); 0.94 (3H, s, 28-H); 0.83 (3H, s, 29-H); 0.97 (3H, s, 30-H); ^13^C-NMR (CDCl_3_): δ 37.2 (C-1); 27.6 (C-2); 79.2 (C-3); 39.0 (C-4); 50.7 (C-5); 24.0 (C-6); 118.2 (C-7); 145.5 (C-8); 48.8 (C-9); 35.0 (C-10); 17.5 (C-11); 31.6 (C-12); 43.5/43.7 (C-13); 50.7 (C-14); 34.2 (C-15); 27.2 (C-16); 45.3 (C-17); 23.2 (C-18); 13.0 (C-19); 45.3/46.4 (C-20); 97.1/102.3 (C-21); 30.2 (C-22); 77.1/78.7 (C-23); 75.1 (C-24); 73.4 (C-25); 26.7 (C-26); 26.7 (C-27); 27.6 (C-28); 14.7 (C-29); 27.3 (C-30).

*Melianodiol* (**6**): colorless amorphous powder; HRESIMS *m/z* 511.3394 [M+Na]^+^ (calcd. for C_30_H_48_O_5_Na, 511.3399; ^1^H-NMR (CDCl_3_): δ 2.73 (1H, td, *J* = 14.5, 5.3 Hz, 2-Hb); 5.21/5.29 (1H, brs, 7-H); 0.81 (3H, s, 18-H); 0.98 (3H, s, 19-H); 5.29 (1H, brs, 21-H); 4.34–4.39/4.45–4.49 (1H, m, 23-H); 3.14/3.20 (1H, brs, 24-H); 1.24 (3H, s, 26-H); 1.26 (3H, s, 27-H); 1.02 (3H, s, 28-H); 1.09 (3H, s, 29-H); 1.00 (3H, s, 30-H); ^13^C-NMR (CDCl_3_): δ 38.5 (C-1); 35.2 (C-2); 216.9 (C-3); 47.9 (C-4); 52.3/52.4 (C-5); 24.3 (C-6); 118.1 (C-7); 145.7 (C-8); 48.4/49.0 (C-9); 34.9 (C-10); 17.7 (C-11); 31.4/31.5 (C-12); 43.5/43.6 (C-13); 50.8 (C-14); 34.2 (C-15); 27.2 (C-16); 45.3 (C-17); 22.5/23.2 (C-18); 12.7 (C-19); 45.3/46.4 (C-20); 97.2/102.2 (C-21); 30.2 (C-22); 77.2/78.7 (C-23); 75.0/75.7 (C-24); 73.4 (C-25); 26.6 (C-26); 26.7 (C-27); 24.5 (C-28); 21.5 (C-29); 27.4 (C-30).

### 3.5. Preparation of Samples for the Adult Immersion Test

Solutions of 0.2% (g/100 mL) for the crude EtOH extract and for the aqueous, hexane, CH_2_Cl_2_, and hydromethanolic phases were prepared using 5% dimethylsulfoxide in distilled water. Each solution was further diluted with water to obtain the 0.2%, 0.1%, and 0.05% concentrations to be used in the adult immersion test. Stock solutions of 0.1% (g/100 mL) obtained from the bioactive hexane extract fractions (A–H) were prepared by dissolving the samples in distilled water containing 5% dimethylsulfoxide. Tests were made with doubly decreasing concentrations of these stock solutions (0.1%, 0.05%, and 0.025%). With compounds **1** and **3**, 0.01% solutions were prepared by dissolving the samples in distilled water containing 5% dimethylsulfoxide. These solutions were subsequently diluted with distilled water to provide three concentrations to be tested (0.01%, 0.005%, and 0.0025%). In a similar fashion, stock solutions of 0.015 g/100 mL of compounds **2** and **4**–**6** in distilled water containing 5% dimethylsulfoxide and 200 µL of Tween 40 were prepared and assayed at concentrations of 0.015%, 0.0075%, and 0.00375%.

### 3.6. Preparation of Ticks

Engorged females of *R. microplus* were obtained from naturally infested Holstein cattle from the Universidade Estadual de Mato Grosso do Sul at Aquidauana. The bovines had been free of commercial acaricidal residues for at least 60 days prior to the experiments. The ticks were collected, cleaned, dried, and selected under a stereomicroscope, based on external morphological conditions and individual biomass (>180 mg), according to Bennet [20]. A total of 1830 ticks were used in the experiment. Each of the three concentrations of each solution sample (extract, phase, or pure compound) was tested in triplicate against 10 pre-weighed ticks (thus totaling 90 ticks per treatment). For the solution controls (distilled water containing 5% dimethylsulfoxide or distilled water containing 5% dimethylsulfoxide and 0.2% Tween 40), three triplicates were used, each containing 10 ticks.

### 3.7. Adult Immersion Test

The engorged females were tested by immersion according to Drummond *et al.* [21]. Treatment and control solutions (10 mL each) were placed in 20 mL beakers. Groups of 10 ticks were weighed and immersed in the designated beaker for 5 min, after which they were dried, placed in Petri dishes, and stored in scotophase at 27 ± 1 °C, RH > 90%, for oviposition. After 15 days, egg biomass was weighed to calculate the percentage of egg conversion (PEC)—*i.e.*, [20]:

[egg mass weight (g)/female weight before treatment (g)] × 100

The eggs were then incubated for a further 15 days, after which period the eggs produced by each group were weighed and incubated until larval eclosion. After mixing with 1 mL of a 1:1 solution of 96% ethanol and glycerin, the larvae and unhatched eggs were counted and the hatching percentage (HP) was calculated as follows:

[number of larvae/(number of larvae + number of eggs)] × 100

The following formulae were used to calculate the estimated reproduction (ER) index and product effectiveness (PE) percentage, according to Drummond *et al.* [21]:

$$\text{ER}=\;\frac{\text{egg}\;\text{weight}\;(\text{g})\;\times \;\%\;\text{hatchability}}{\text{weight}\;\text{of}\;\text{females}\;(\text{g})}\;\times \;20,000\;*\;*\;\text{Constant}\;\text{expressing}\;\text{the}\;\text{number}\;\text{of}\;\text{eggs}\;\text{in}\;1\;\text{g}.$$

$$\text{PE}\;=\;\frac{\text{ER}\;(\text{control}\;\text{group})\;-\;\text{ER}\;(\text{treated}\;\text{group})}{\text{ER}\;(\text{control}\;\text{group})}\;\times 100$$

### 3.8. Statistical Analysis

One-way ANOVA (α = 0.05) and Tukey *post-hoc* comparisons (*p* < 0.05) were performed to address whether the samples investigated and related variables (*i.e.*, concentrations and percentages for each fraction and substance) exhibited biocontrol activity against engorged females. Normality and homogeneity were assessed by performing Kolmogorov–Smirnov and Levene tests (α > 0.10), respectively. PEC, HP, and PE values were expressed as means ± SD.

## 4. Conclusions

This bioassay-guided fractionation of the hexane phase obtained by partitioning the ethanol extract of *Guarea kunthiana* fruits led to the isolation of a new protolimonoid, 3β-*O*-tigloylmelianol, that proved remarkably active against engorged females of *R. (B) microplus*. Melianone, isolated from the same phase, was devoid of any significant activity in the adult immersion test.

This is the first report on the isolation, employing bioassay-guided fractionation of a plant extract, of a protolimonoid active against engorged females of this cattle tick. Other protolimonoids (melianol, meliantriol, melianodiol, and the new 3β-*O*-tigloylmeliantriol) were isolated from the dichloromethane phase, but these exhibited unremarkable results in this test. This phase was also shown to contain the limonoids humilinolide E, methyl 2-hydroxy-3β-tigloyloxy-1-oxomeliac-8(30)-enate, swietenine acetate, and methyl angolensate.

The results obtained revealed 3β-*O*-tigloylmelianol to be a promising candidate for the development of a biocontrol agent against engorged females of *R. (B.) microplus*, as an alternative to environmentally hazardous synthetic acaricides, particularly those against which this cattle tick has developed resistance.

## Supplementary Materials

Supplementary materials can be accessed at: http://www.mdpi.com/1420-3049/20/01/0111/s1.

## References

[1] Guglielmone A.A. (1995). Epidemiology of babesiosis and anaplasmosis in South and Central America. Vet. Parasitol..

[2] Giles J.R., Peterson A.T., Busch J.D., Olafson P.U., Scoles G.A., Davey R.B., Pound J.M., Kammlah D.M., Lohmeyer K.H., Wagner D.M. (2014). Invasive potential of cattle fever ticks in the southern United States. Parasit. Vectors.

[3] Rodríguez-Vivas R.I., Perez-Cogollo L.C., Rosado-Aguilar J.A., Ojeda-Chi M.M., Trinidad-Martinez I., Miller R.J., Li A.Y., Leon A.P., Guerrero F., Klafke G. (2014). *Rhipicephalus* (*Boophilus*) *microplus* resistant to acaricides and ivermectin in cattle farms of Mexico. Rev. Bras. Parasitol. Vet..

[4] Borges L.M.F., Sousa L.A.D., Barbosa C.S. (2011). Perspectives for the use of plant extracts to control the cattle tick *Rhipicephalus* (*Boophilus*) *microplus*. Rev. Bras. Parasitol. Vet..

[5] Grisi L., Leite R.C., Martins J.R.S., Barros A.T.M., Andreotti R., Cançado P.H.D., León A.A.P., Pereira J.P., Villela H.S. (2014). Reassessment of the potential economic impact of cattle parasites in Brazil. Rev. Bras. Parasitol. Vet..

[6] Foil L.D., Coleman P., Eisler M., Fragoso-Sanchez H., Garcia-Vazquez Z., Guerrero F.D., Jonsson N.N., Langstaff I.G., Li A.Y., Machila N. (2004). Factors that influence the prevalence of acaricide resistance and tick-borne diseases. Vet. Parasitol..

[7] Mendes M.C., Lima C.K.P., Nogueira A.H.C., Yoshihara E., Chiebao D.P., Gabriel F. H.L., Ueno T.E.H., Namindome A., Klafke G.M. (2011). Resistance to cypermethrin, deltamethrin, and chlorpyriphos in populations of *Rhipicephalus (Boophilus) microplus* (Acari:Ixodidae) from small farms of the State of São Paulo, Brazil. Vet. Parasitol..

[8] Mendes M.C., Duarte F.C., Martins J.R., Klafke G.M., Fiorini L.C., Barros A.T.M. (2013). Characterization of the pyrethroid resistance profile of *Rhipicephalus (Boophilus) microplus* populations from the states of Rio Grande do Sul and Mato Grosso do Sul, Brazil. Rev. Bras. Parasitol. Vet..

[9] Semmler M., Abdel-Ghaffar F., Al-Rasheid K., Mehlhorn H. (2009). Nature helps: From research to products against blood-sucking arthropods. Parasitol. Res..

[10] Barbosa C.S., Borges L.M.F., Nicácio J., Alves R.D., Miguita C.H., Violante I.M.P., Hamerski L., Garcez W.S., Garcez F. R. (2013). *In vitro* activities of plant extracts from the Brazilian Cerrado and Pantanal against *Rhipicephalus (Boophilus) microplus* (Acari: Ixodidae). Exp. Appl. Acarol..

[11] Abbas R.Z., Zaman M.A., Colwell D.D., Gilleard J., Iqbal Z. (2014). Acaricide resistance in cattle ticks and approaches to its management: the state of play. Vet. Parasitol..

[12] Nakanishi T., Inada A., Lavie D. (1986). A new tirucallane-type triterpenoid derivative, lipomelianol from fruits of *Melia toosendan* Sieb et Zucc. Chem. Pharm. Bull..

[13] Joseph-Nathan P., Wesener J.R., Günther H. (1984). A two-dimensional NMR study of angelic and tiglic acid. Org. Magn. Reson..

[14] Ntalli N.G., Cottiglia F., Bueno C.A., Alché L.E., Leonti M., Vargiu S., Bifulco E., Menkissoglu-Spiroudi U., Caboni P. (2010). Cytotoxic tirucallane triterpenoids from *Melia azedarach* fruits. Molecules.

[15] Lins A.P., Braggio M.M., Felicio J.D., Giuratti A.M., Felicio J.C. (1992). Chemical and pharmacological aspects of *Guarea guidona*. Rev. Latinoam. Quím..

[16] Jimenez A., Villareal C., Toscano R.A., Cook M., Arnason J.T., Bye R., Mata R. (1998). Limonoids from *Swietenia humilis* and *Guarea grandiflora* (Meliaceae). Phytochemistry.

[17] Kurimoto S., Takaishi Y., Ahmed F.A., Kashiwada Y. (2014). Triterpenoids from the fruits of *Azadirachta indica* (Meliaceae). Fitoterapia.

[18] Puripattanavong J., Weber S., Brecht V., Frahm A.W. (2000). Phytochemical investigation of *Aglaia andamanica*. Planta Med..

[19] Miguita C.H., Sarmento U.C., Hamerski L., Garcez W.S., Garcez F.R. (2014). Mexicanolide- and andirobine-type limonoids from the fruits of *Guarea kunthiana*. Rec. Nat. Prod..

[20] Bennett G. F. (1974). Oviposition of *Boophilus microplus* (Canestrini) (Acarida Ixodidae). I. Influence of tick size on egg production. Acarologia.

[21] Drummond R. O., Ernest S. E., Trevino J. L., Gladney W. J., Graham O. H. (1973). *Boophilus microplus annulatus* and *Boophilus microplus*: Laboratory tests for insecticides. J. Econ. Entomol..

