# Supplementary material for: 3β-O-Tigloylmelianol from Guarea kunthiana: A New Potential Agent to Control Rhipicephalus (Boophilus) microplus, a Cattle Tick of Veterinary Significance
Source: Molecules. 2014 Dec 23;20(1):111–26. doi: 10.3390/molecules20010111 (PMC6272232; doi:10.3390/molecules20010111)
Supplement: Supplementary file 1 [file molecules-20-00111-s001.pdf]

Supplementary Materials

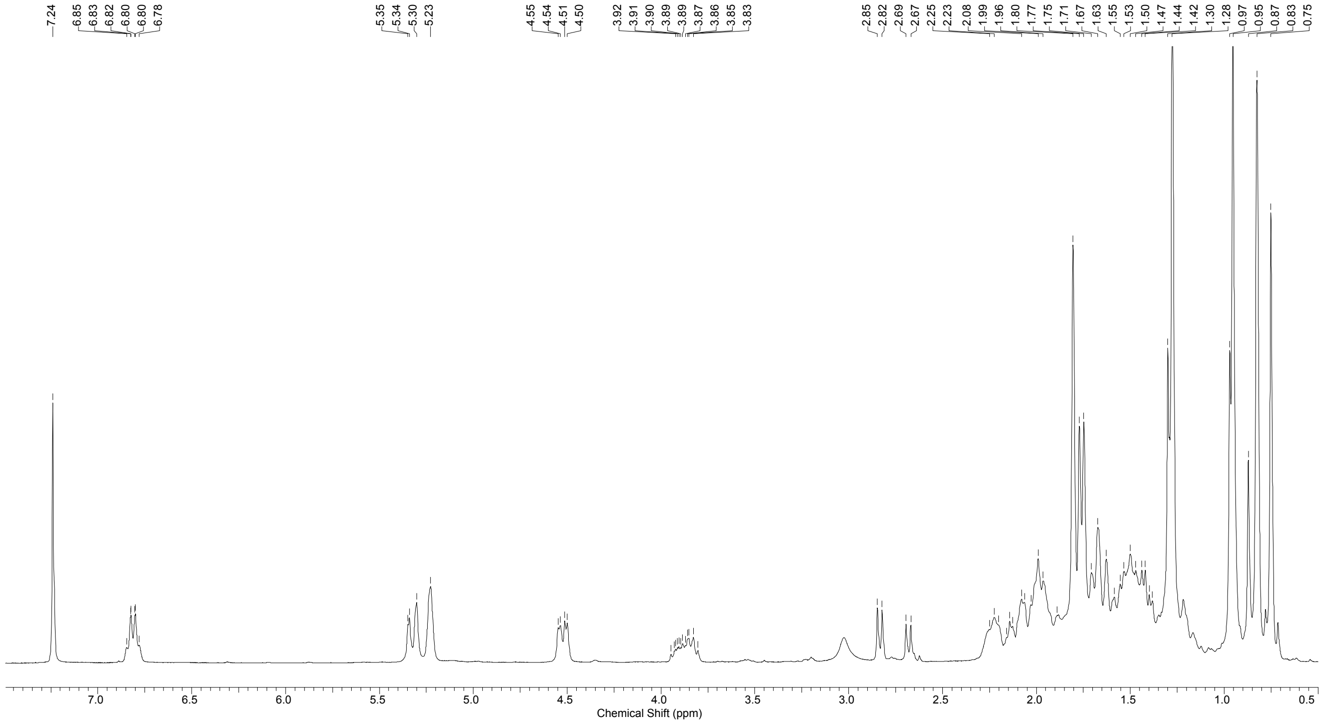

Figure S1. ^{1}H-NMR spectrum (300 MHz, $CDCl_3$) of **1**.

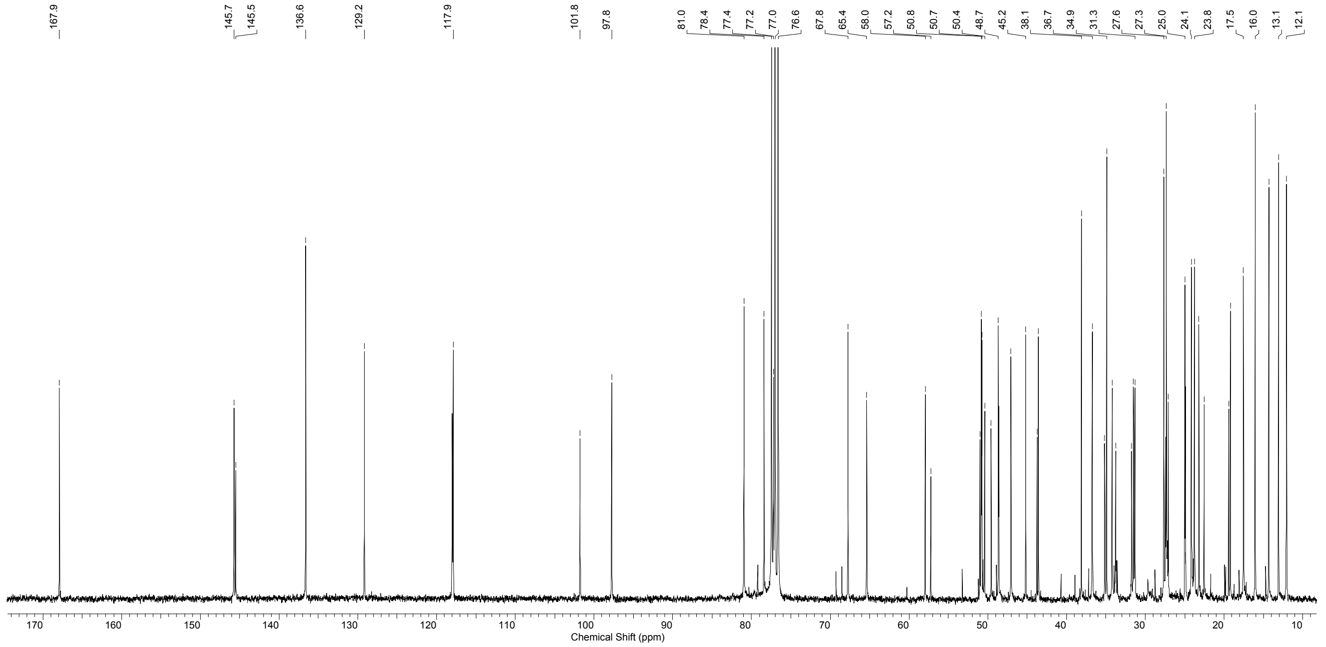

Figure S2. ^{13}C-NMR spectrum (75 MHz, $CDCl_3$) of **1**.

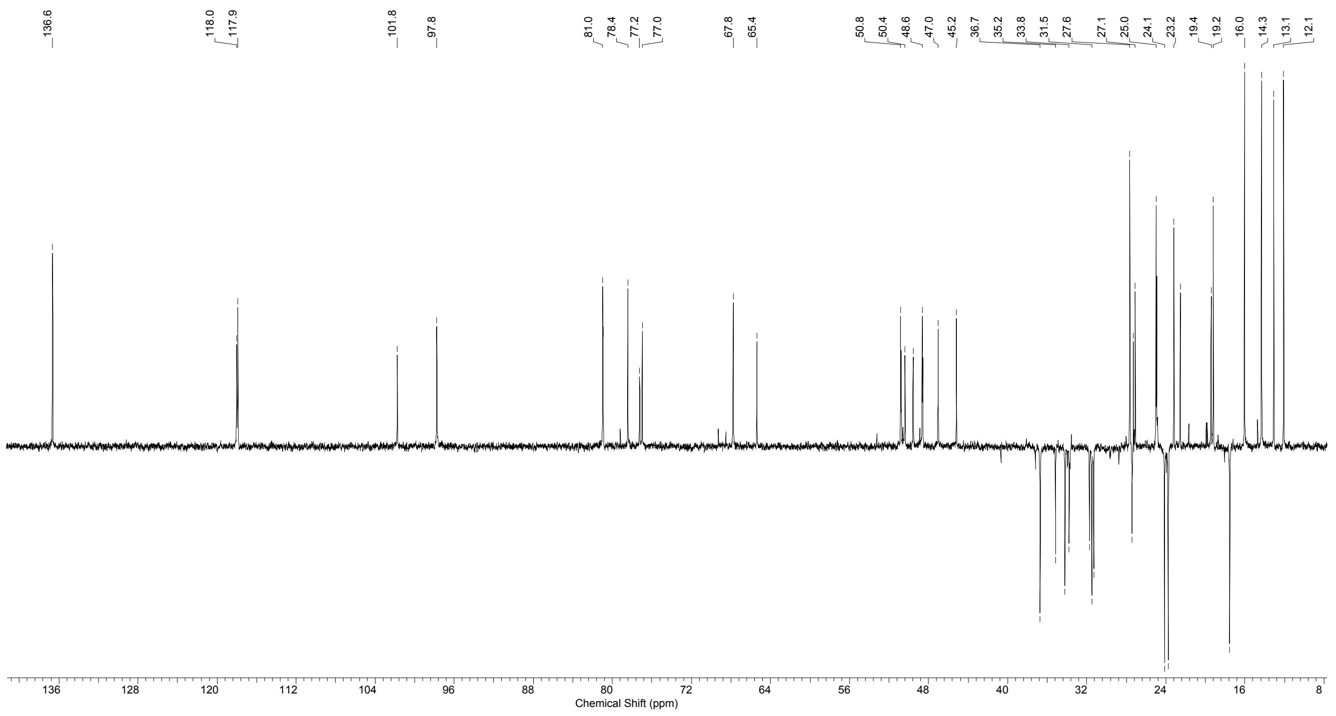

Figure S3. NMR DEPT 135 spectrum (75 MHz, $CDCl_3$) of **1**.

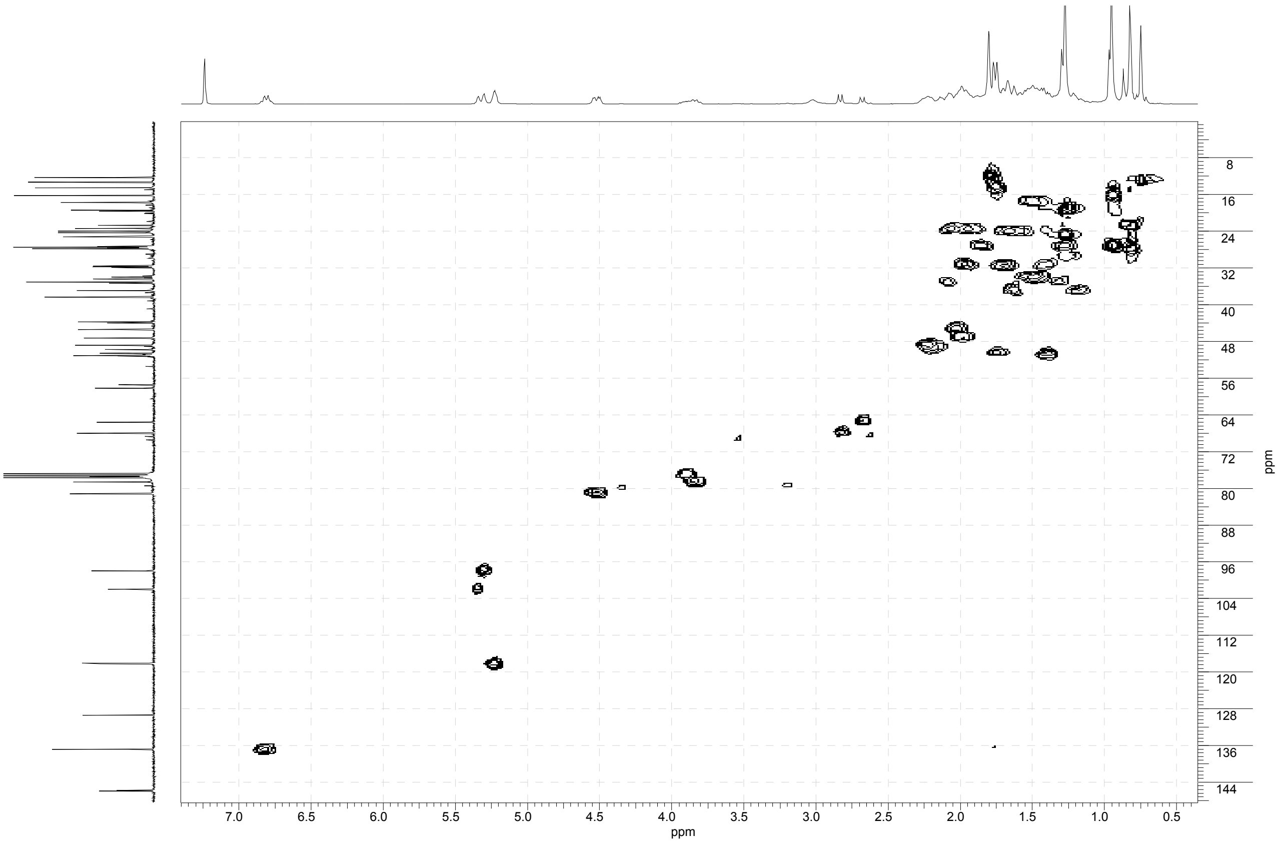

Figure S4. HSQC (300/75 MHz, $CDCl_3$) of **1**.

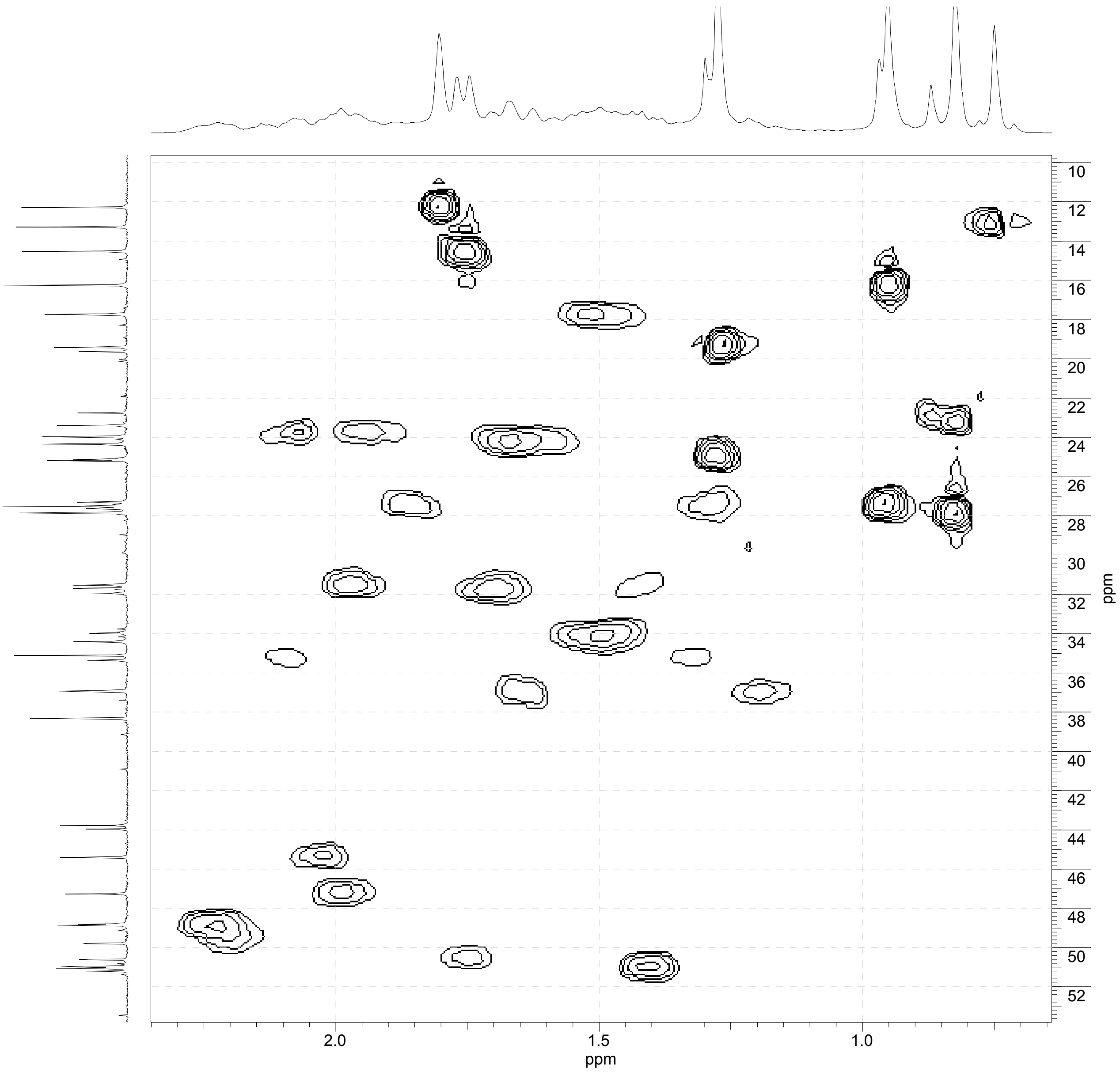

Figure S5. HSQC (300/75 MHz, $CDCl_3$) [Expansion] of **1**.

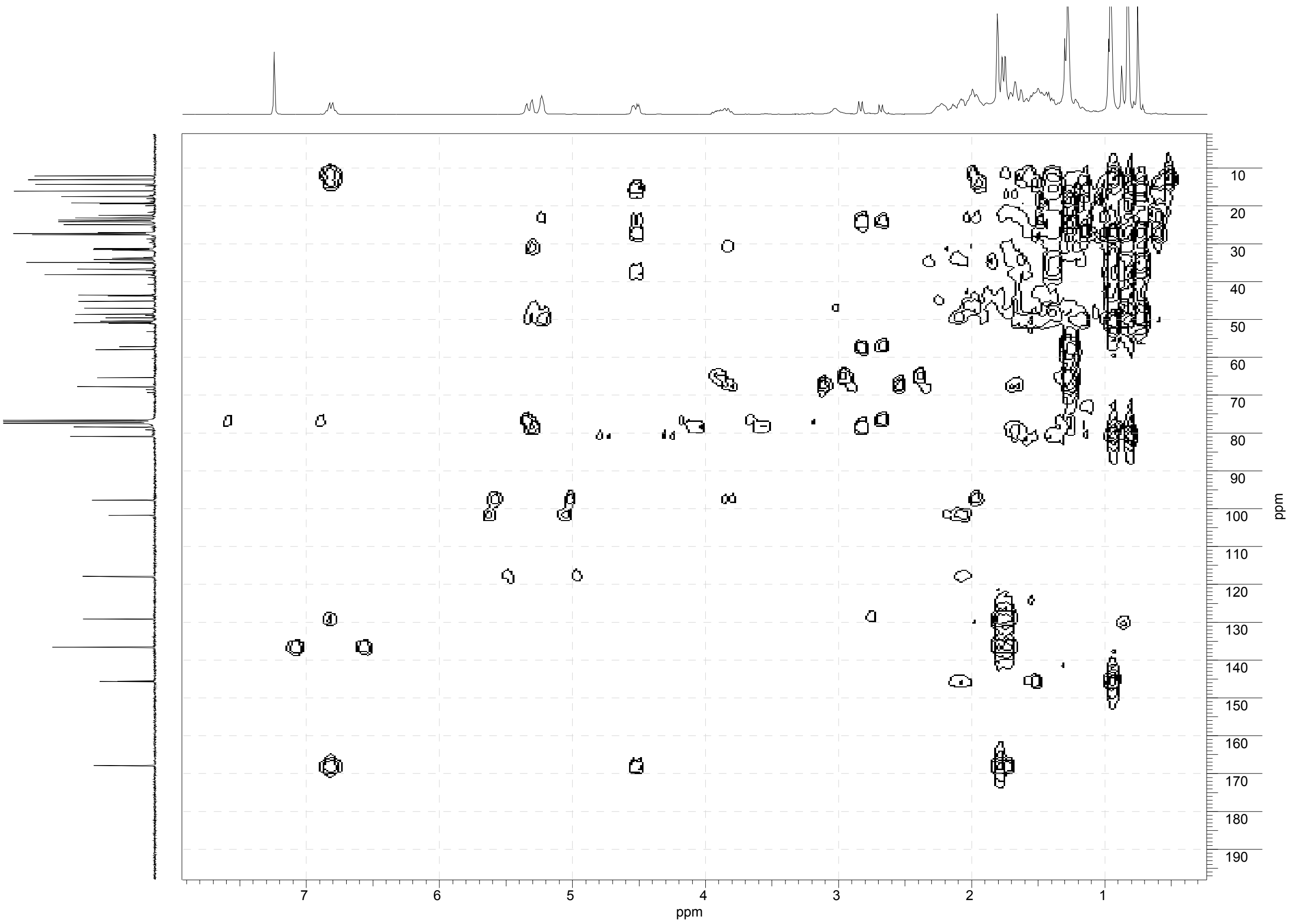

Figure S6. HMBC (300/75 MHz,$CDCl_3$) of **1**.

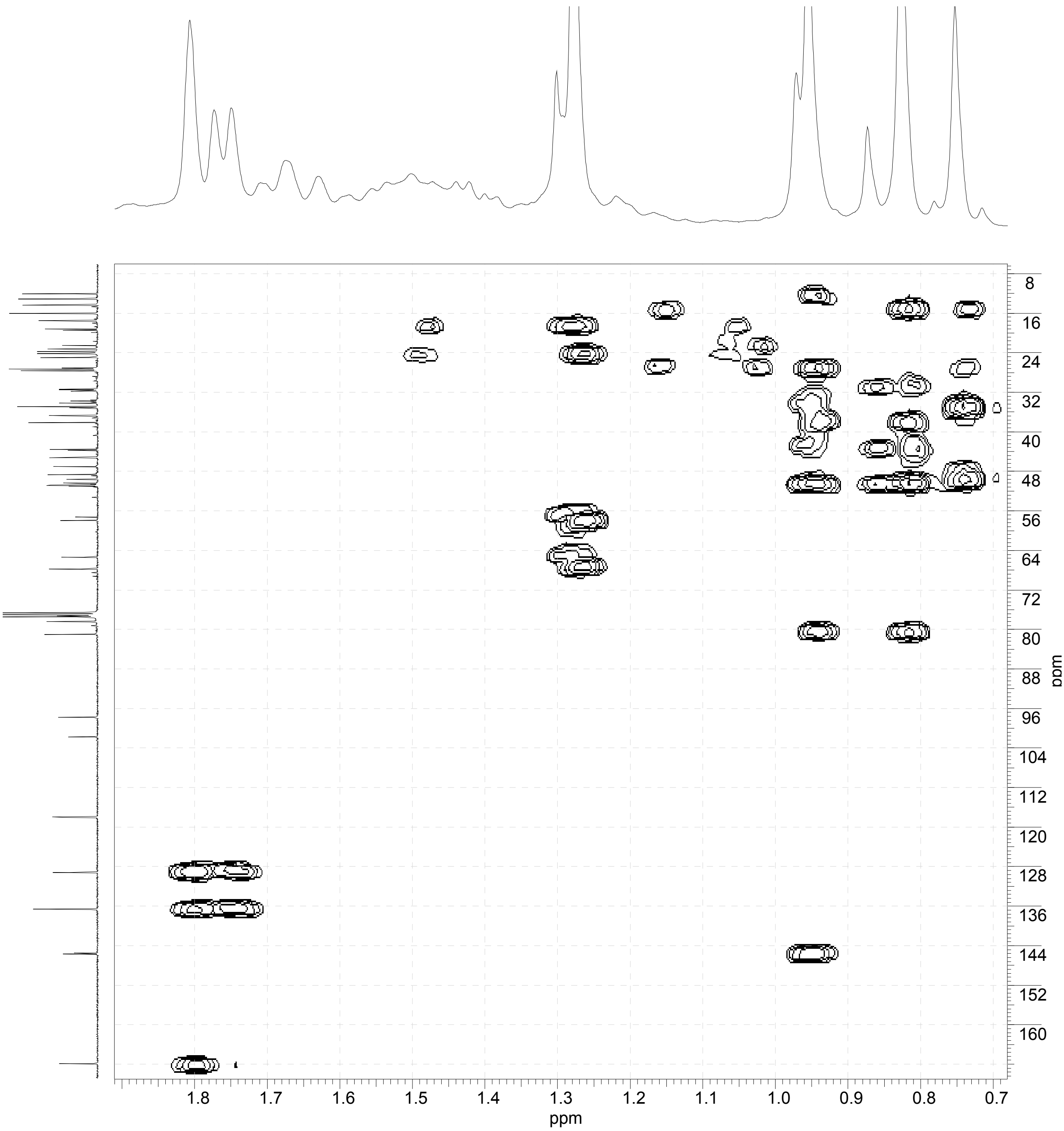

Figure S7. HMBC (300/75 MHz, $CDCl_3$) [Expansion] of **1**.

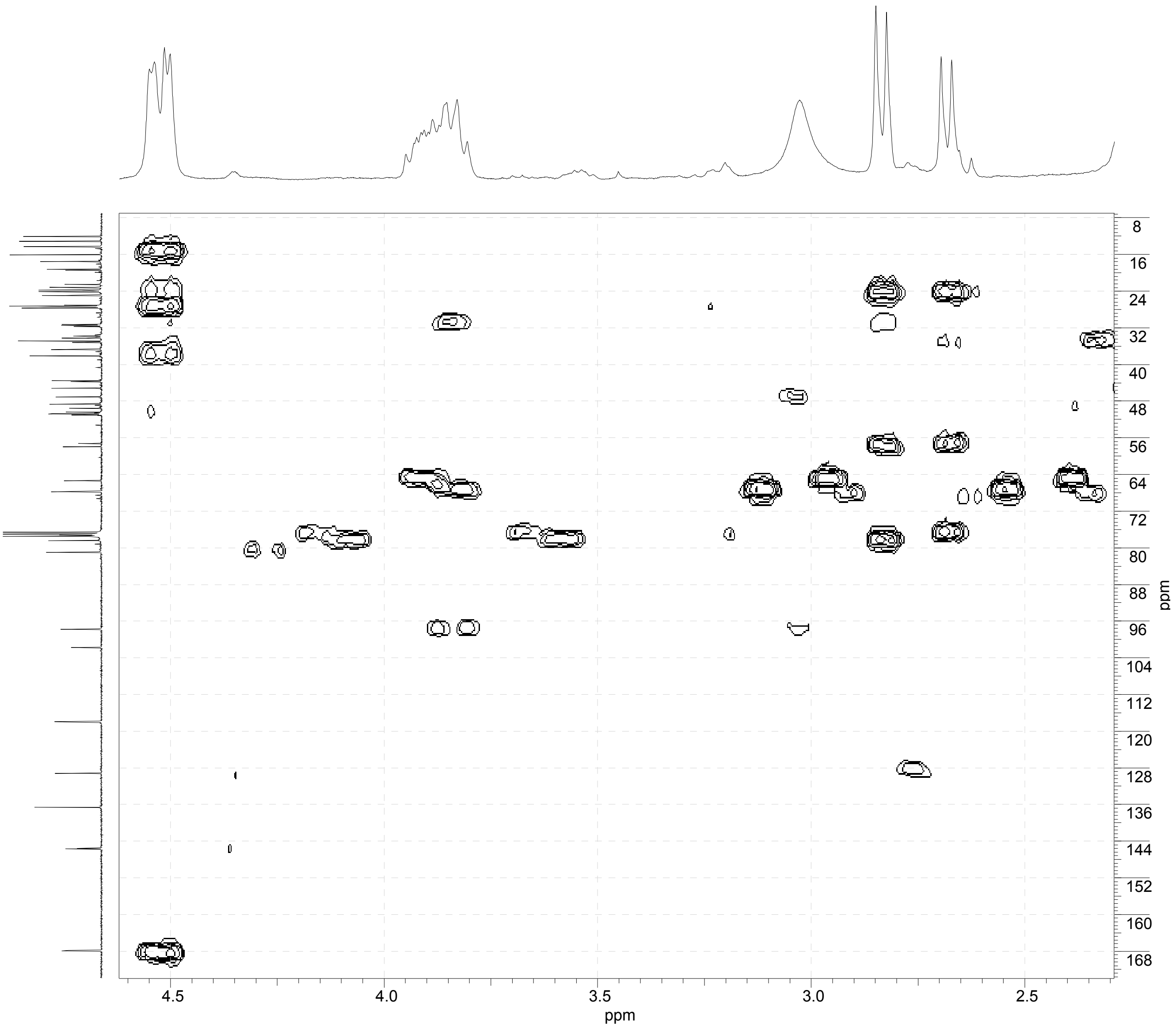

Figure S8. HMBC (300/75 MHz, $CDCl_3$) [Expansion] of **1**.

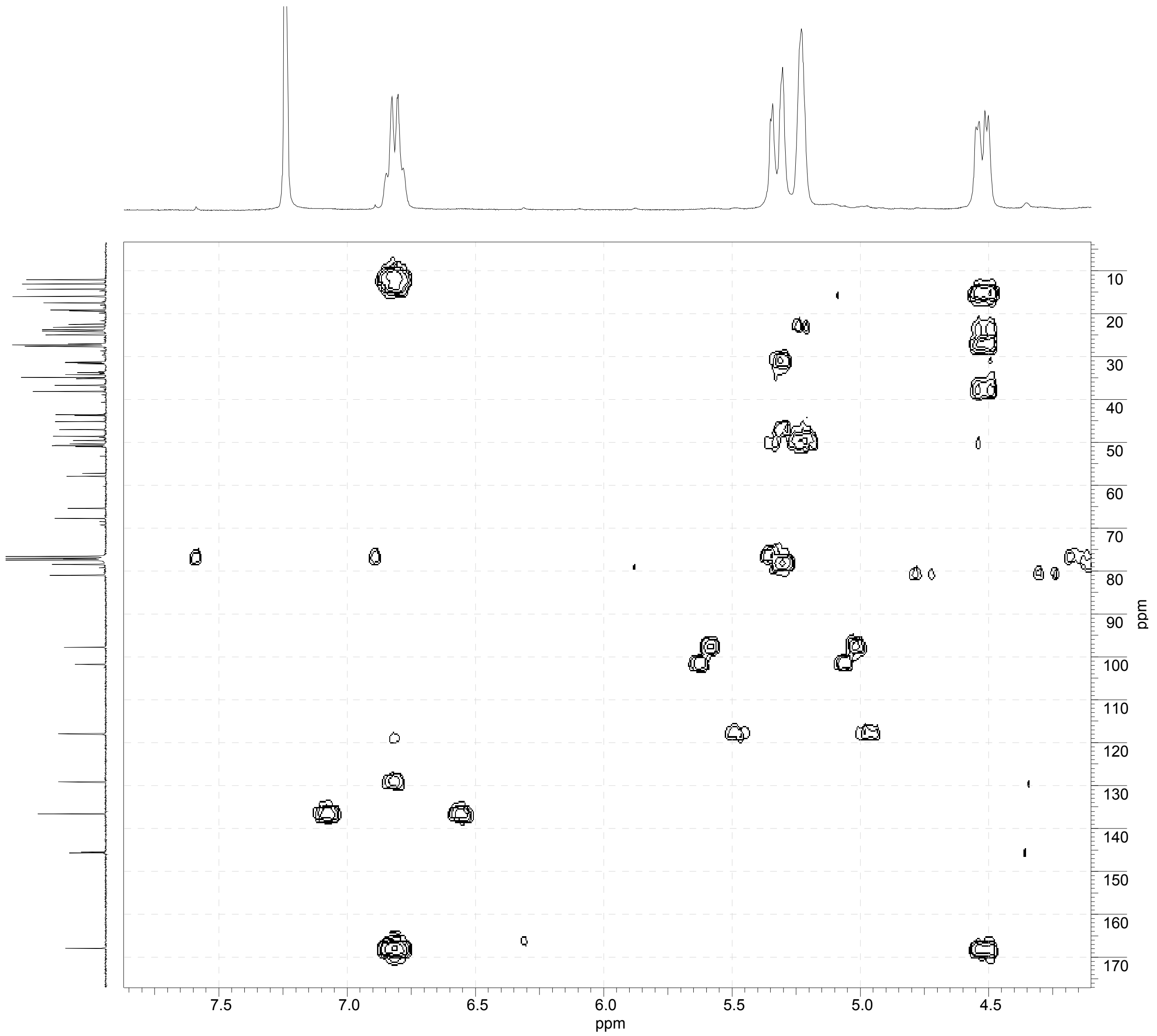

Figure S9. HMBC (300/75 MHz, $CDCl_3$) [Expansion] of **1**.

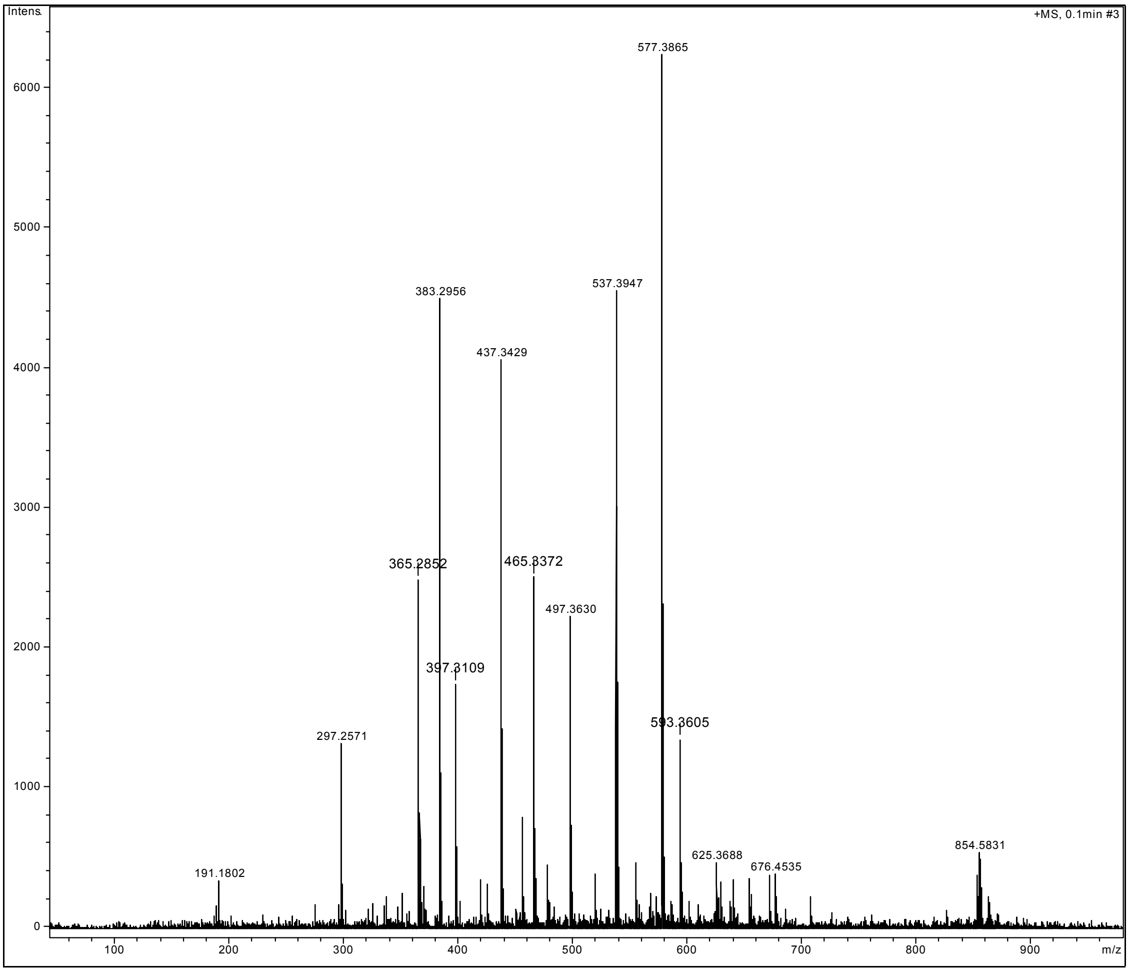

Figure S10. HRESIMS spectrum (positive mode) of **1**.

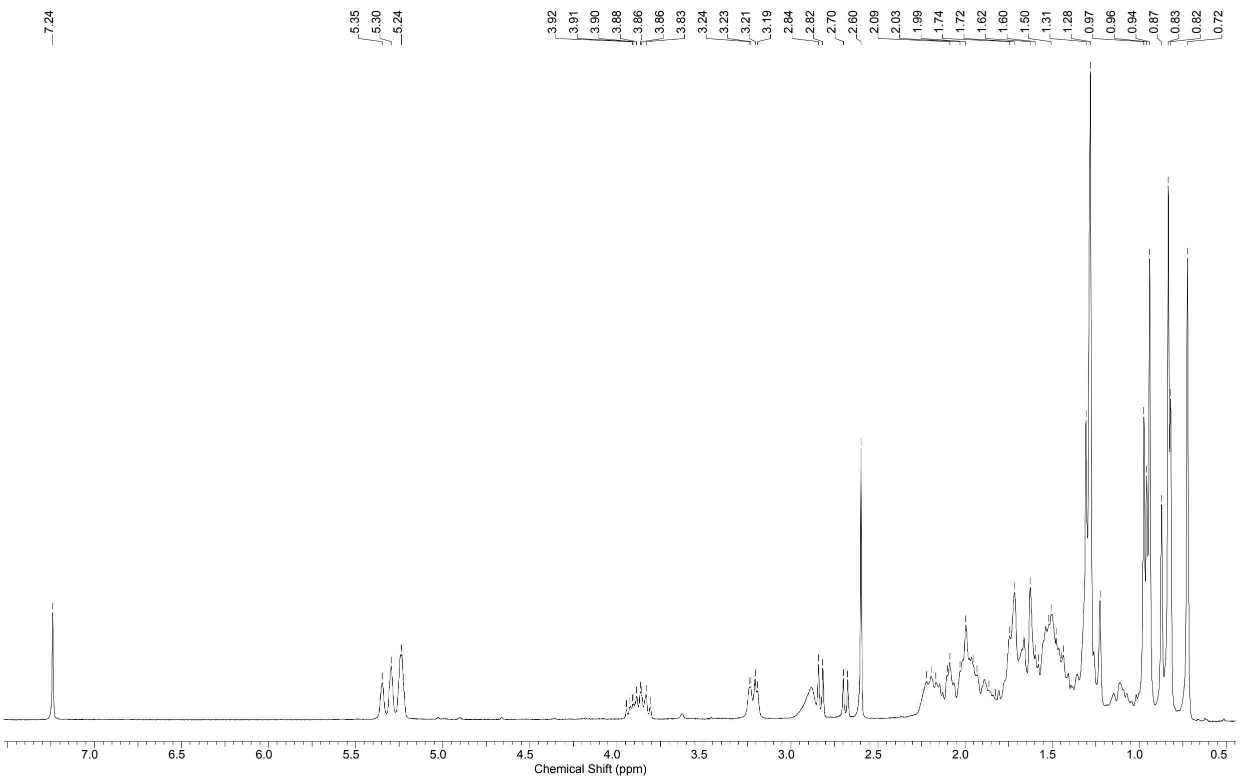

Figure S11. ^{1}H-NMR spectrum (300 MHz, $CDCl_3$) of **2**.

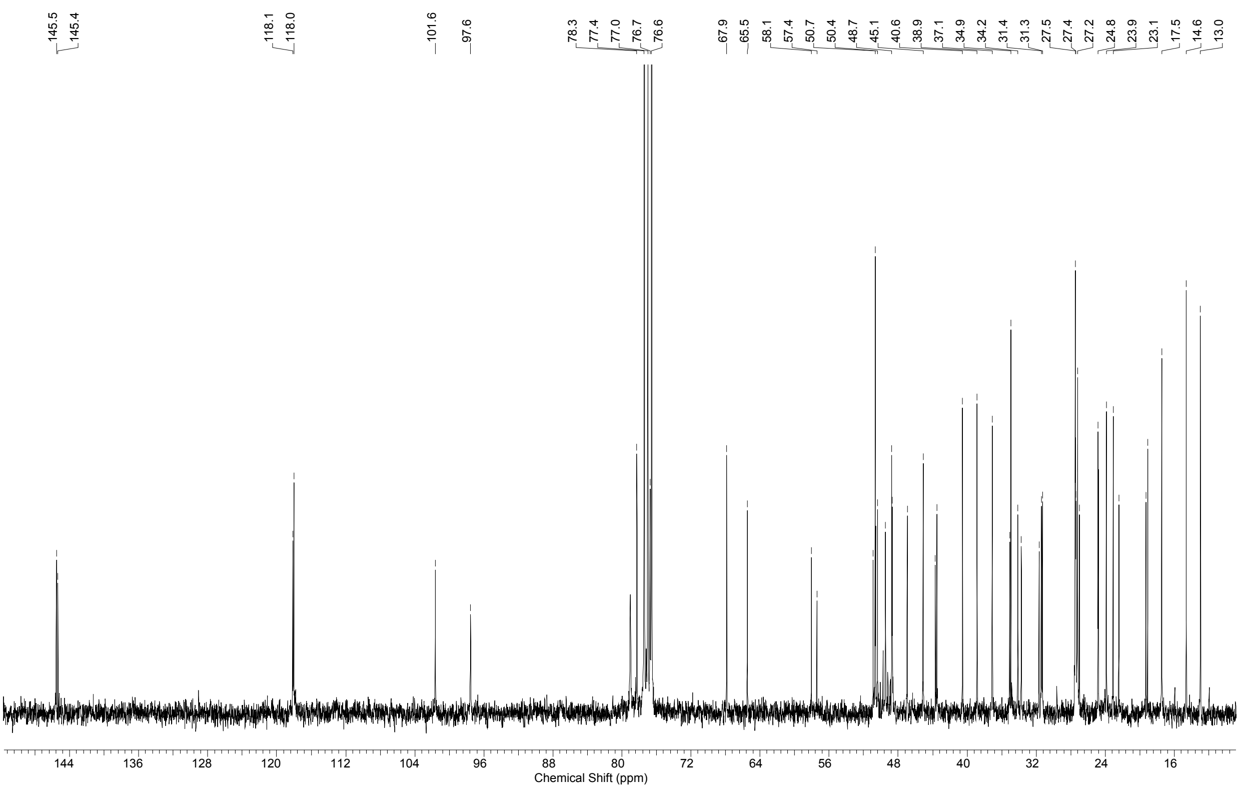

Figure S12. ^{13}C-NMR spectrum (75 MHz, $CDCl_3$) of **2**.

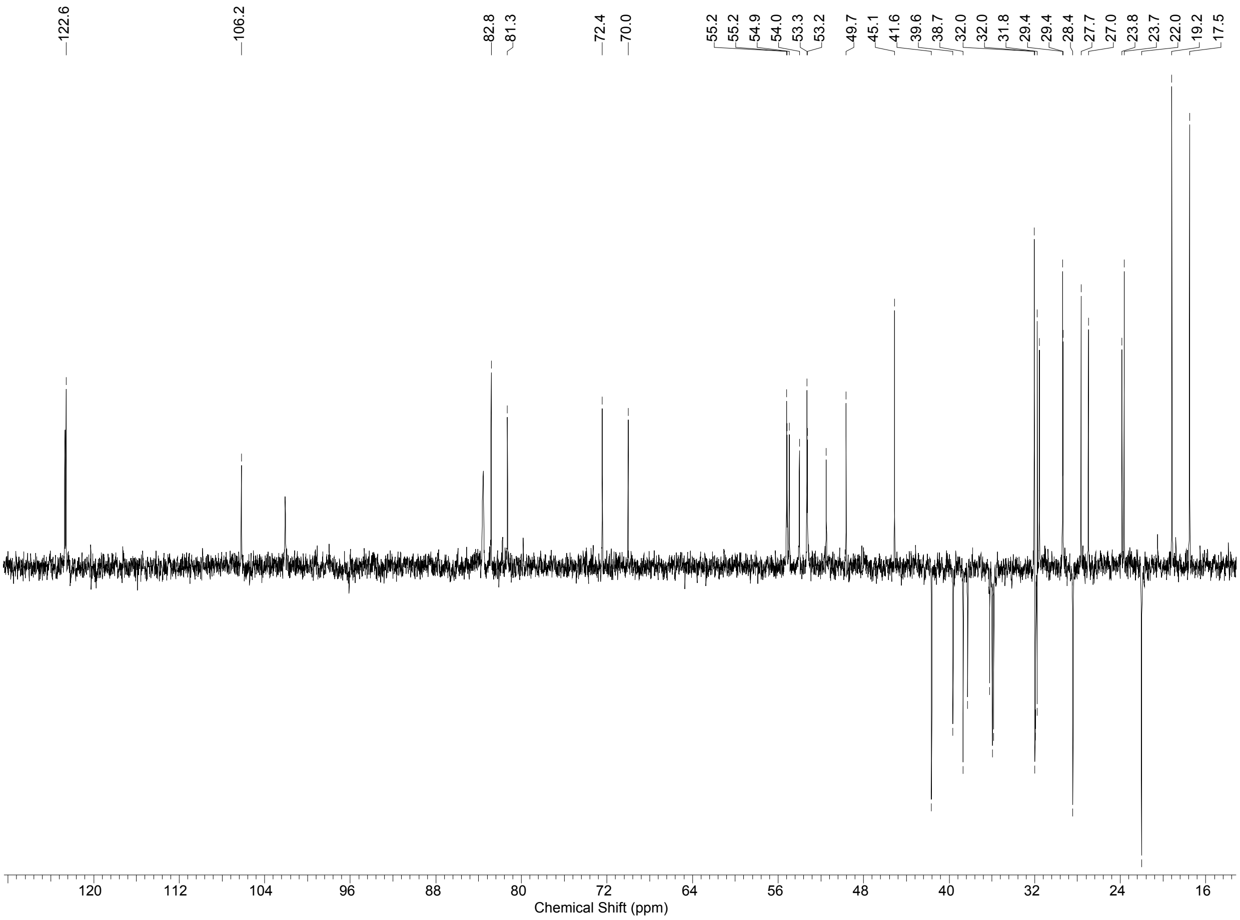

Figure S13. NMR DEPT 135 spectrum (75 MHz, $CDCl_3$) of **2**.

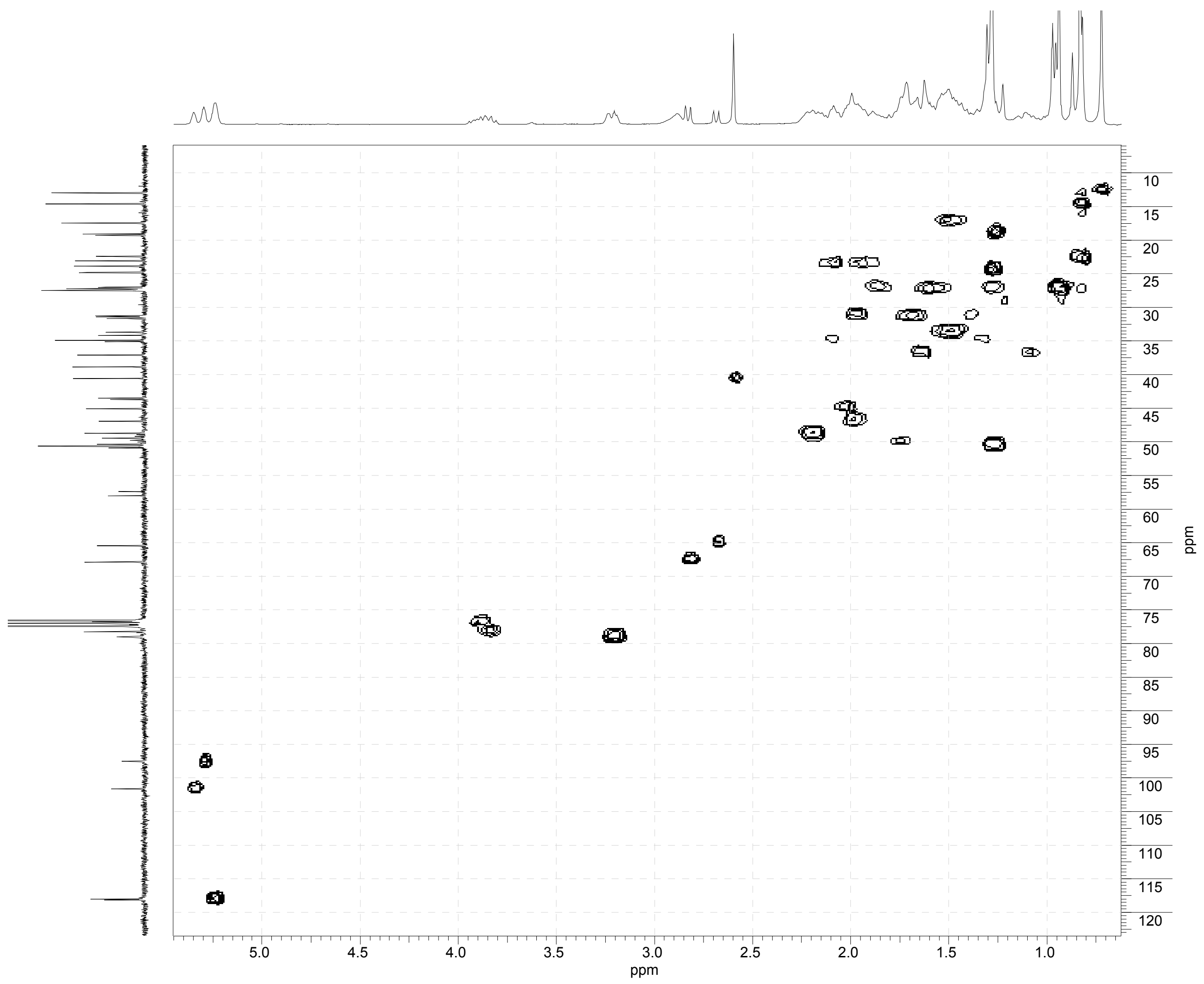

Figure S14. HSQC (300/75 MHz, $CDCl_3$) of **2**.

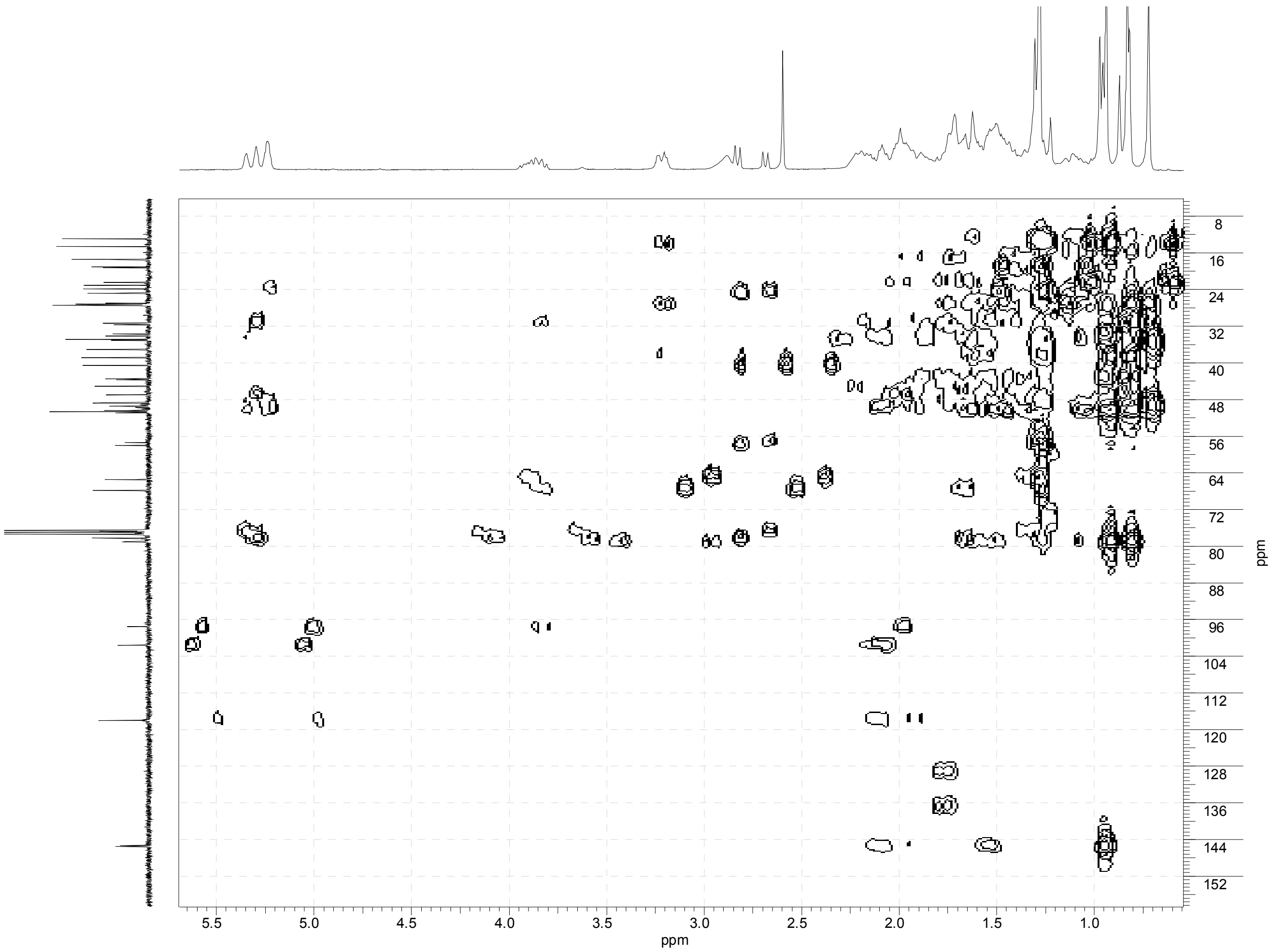

Figure S15. HMBC (300/75 MHz, $CDCl_3$) of **2**.

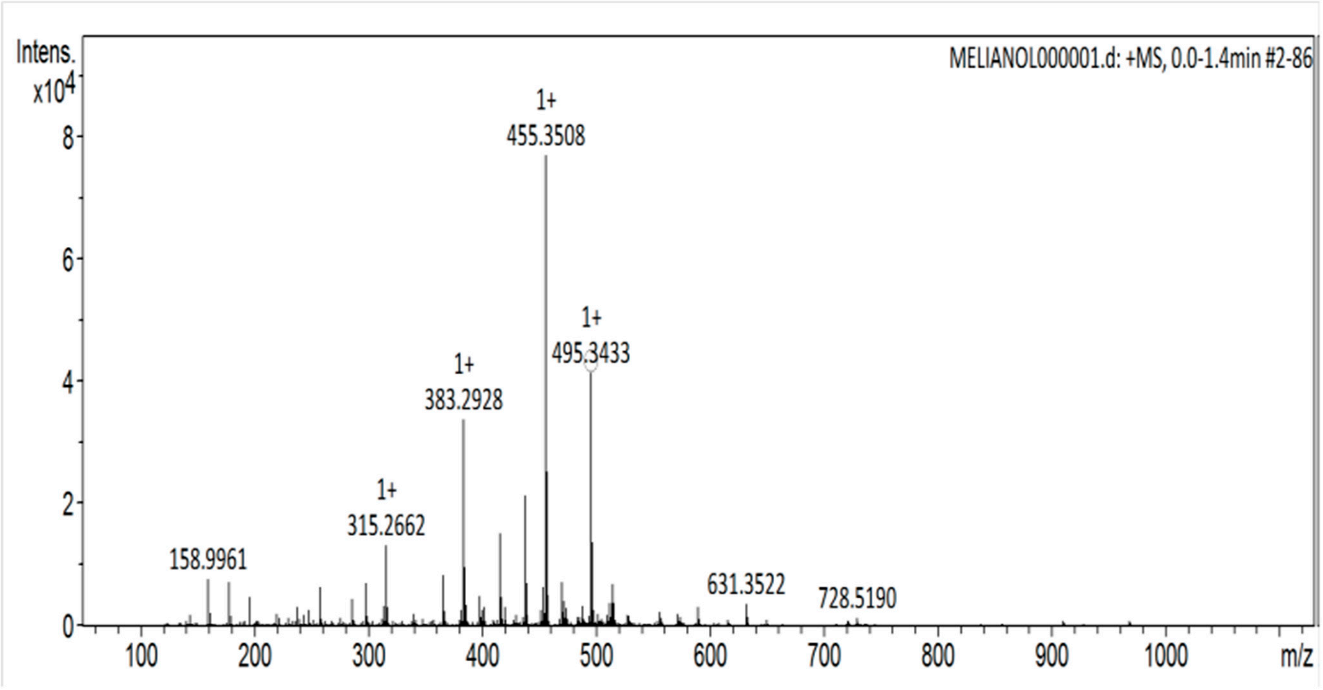

Figure S16. HRESIMS spectrum (positive mode) of **2**.

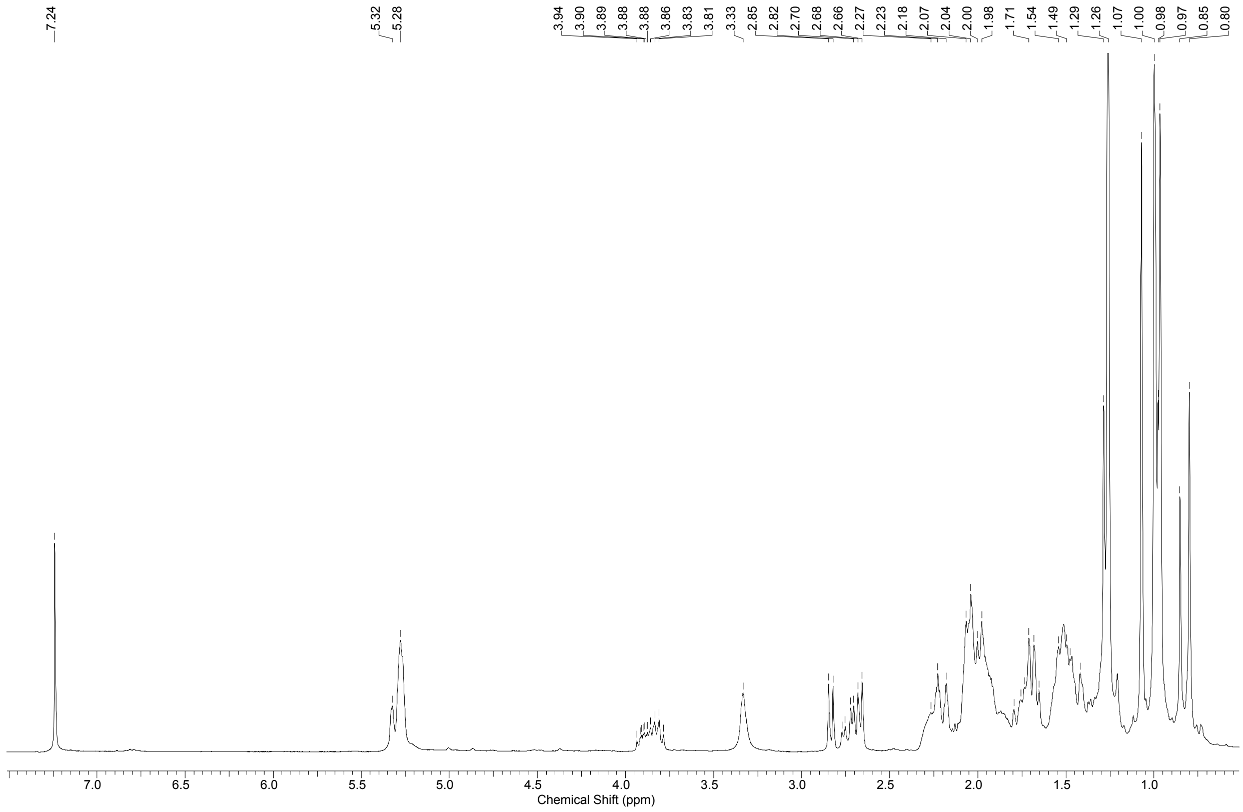

Figure S17. ^{1}H-NMR spectrum (300 MHz, $CDCl_3$) of **3**.

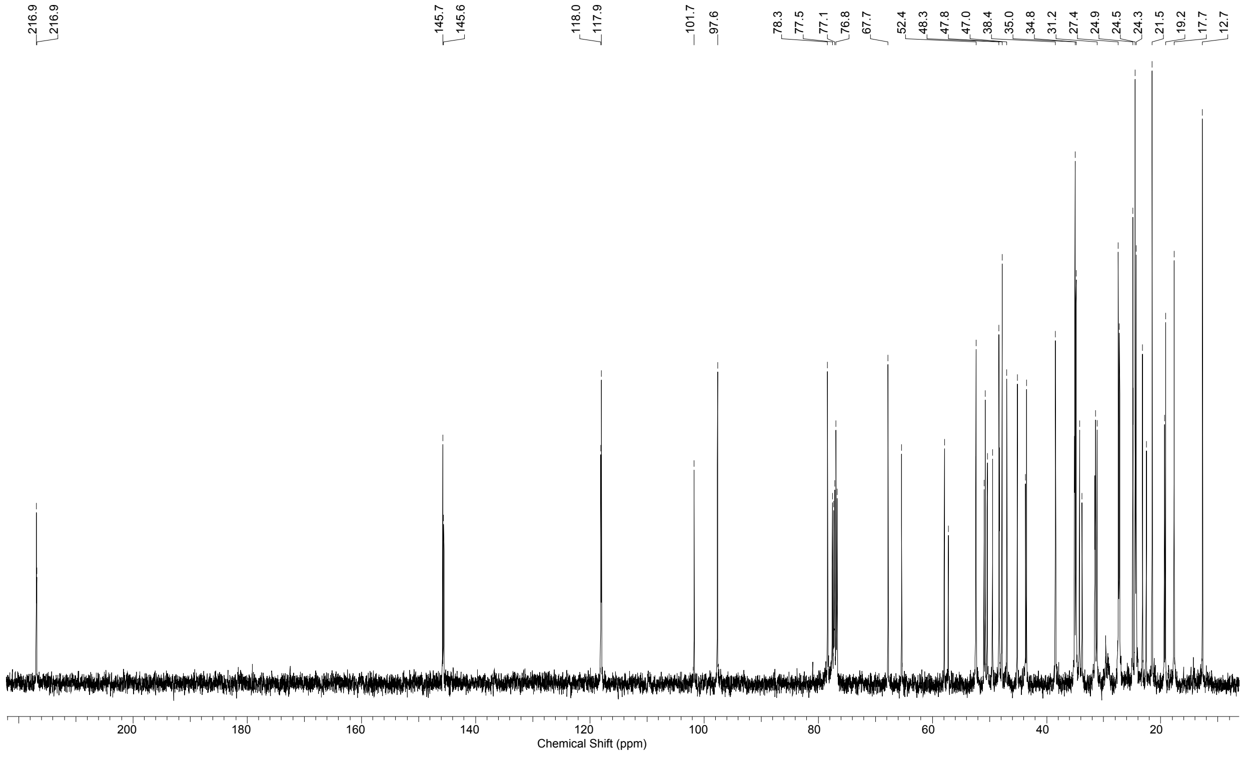

Figure S18. ^{13}C-NMR spectrum (75 MHz, $CDCl_3$) of **3**.

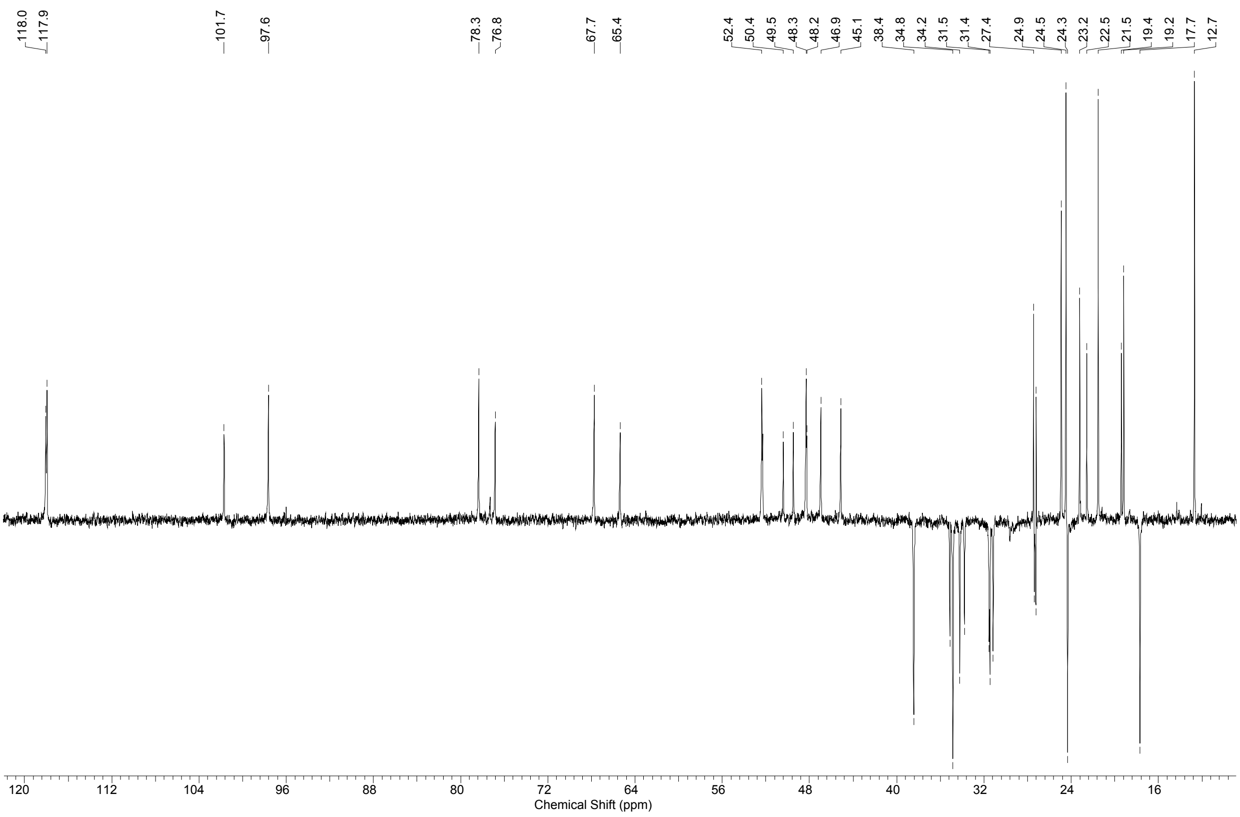

Figure S19. NMR DEPT 135 spectrum (75 MHz, $CDCl_3$) of **3**.

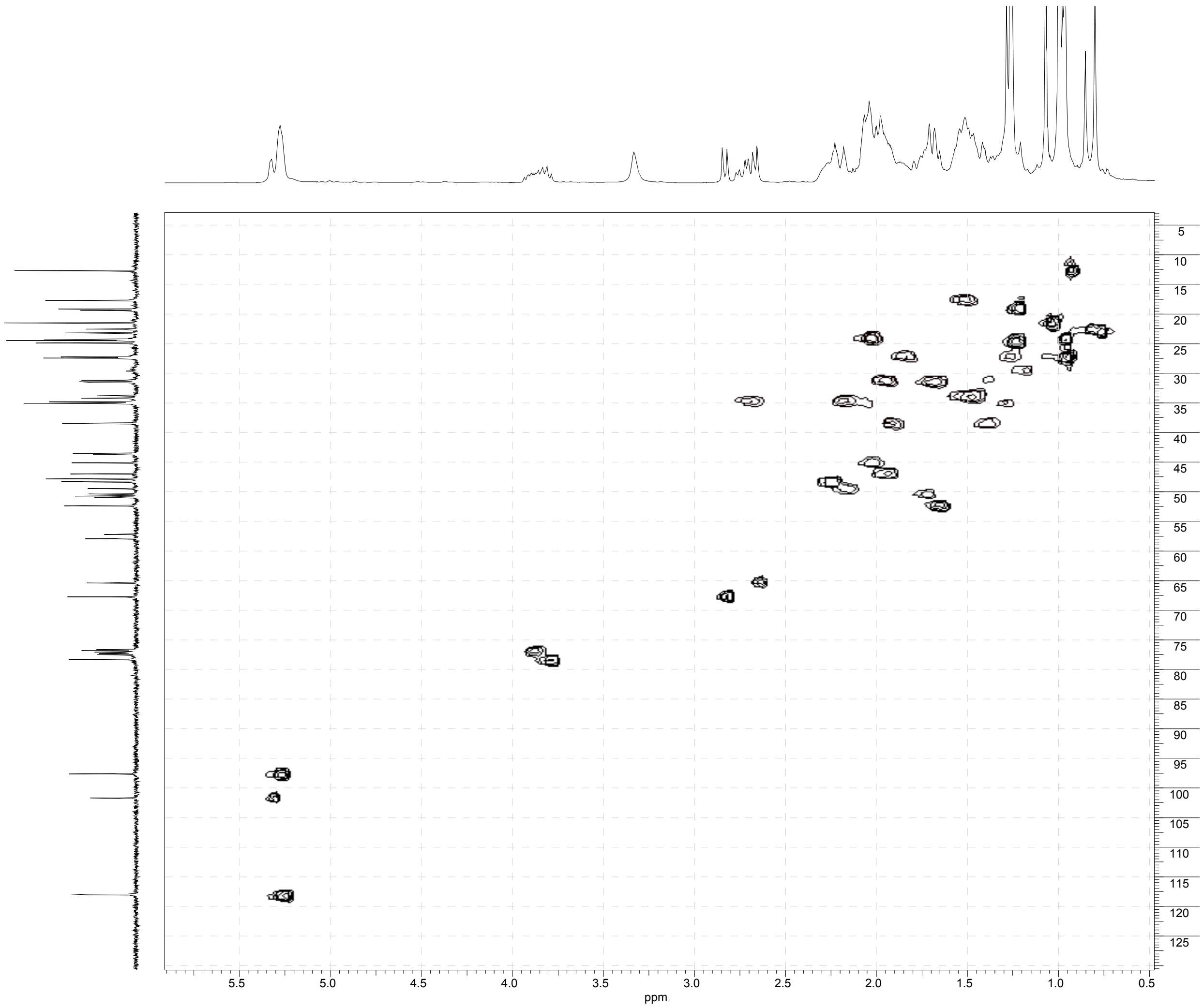

Figure S20. HSQC (300/75 MHz, $CDCl_3$) of **3**.

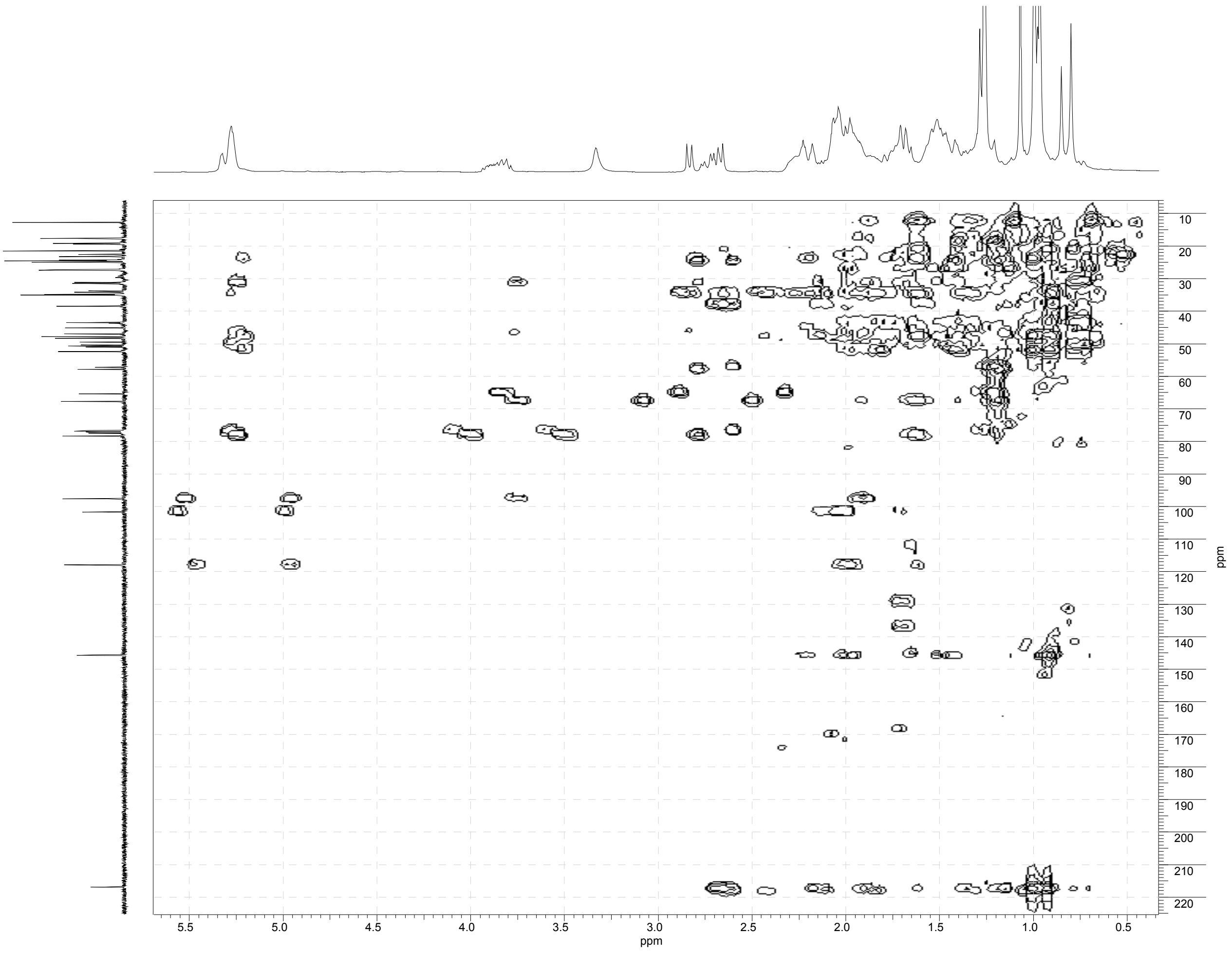

Figure S21. HMBC (300/75 MHz, $CDCl_3$) of **3**.

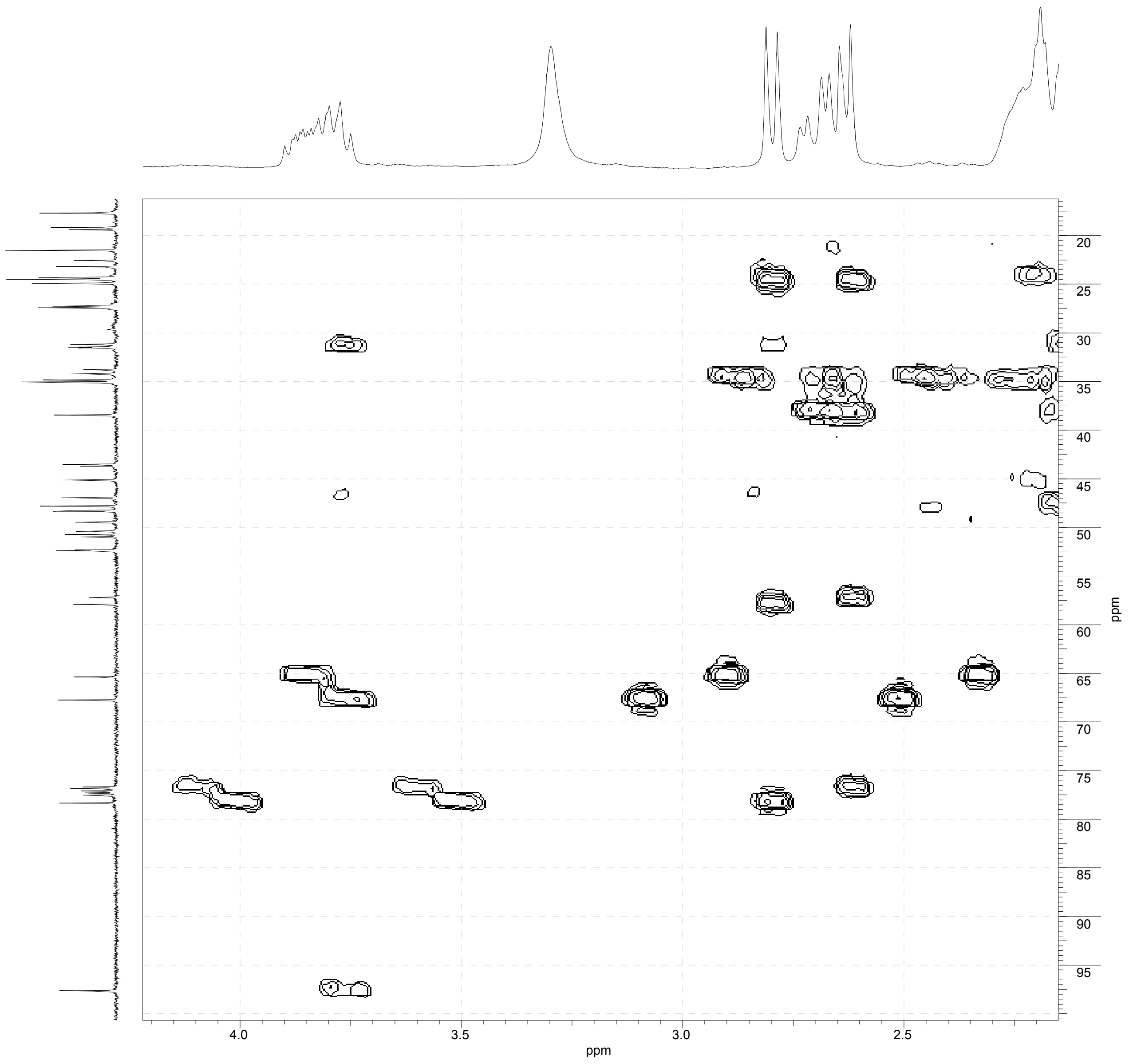

Figure S22. HMBC (300/75 MHz, $CDCl_3$) [Expansion] of **3**.

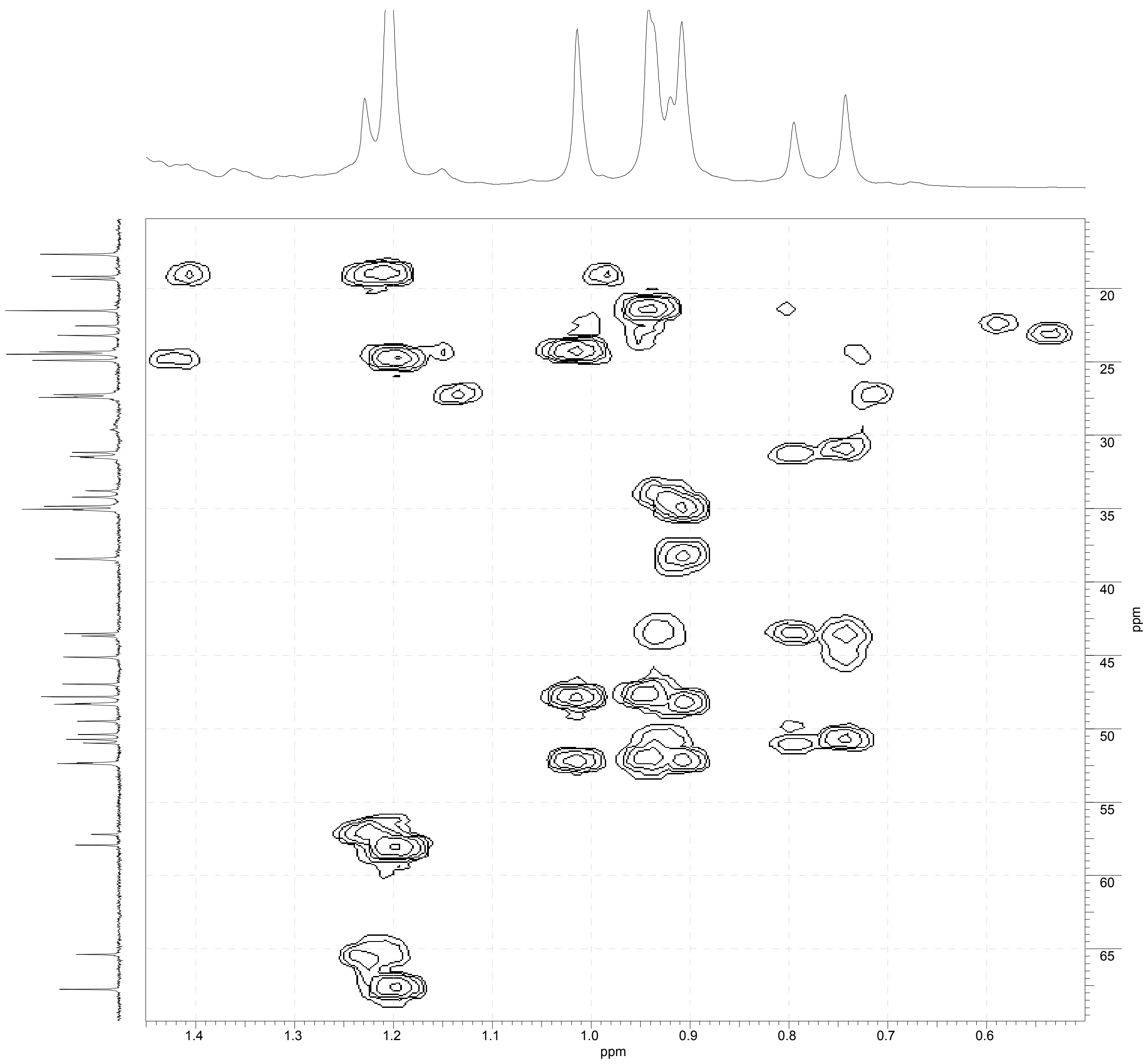

Figure S23. HMBC (300/75 MHz, $CDCl_3$) [Expansion] of **3**.

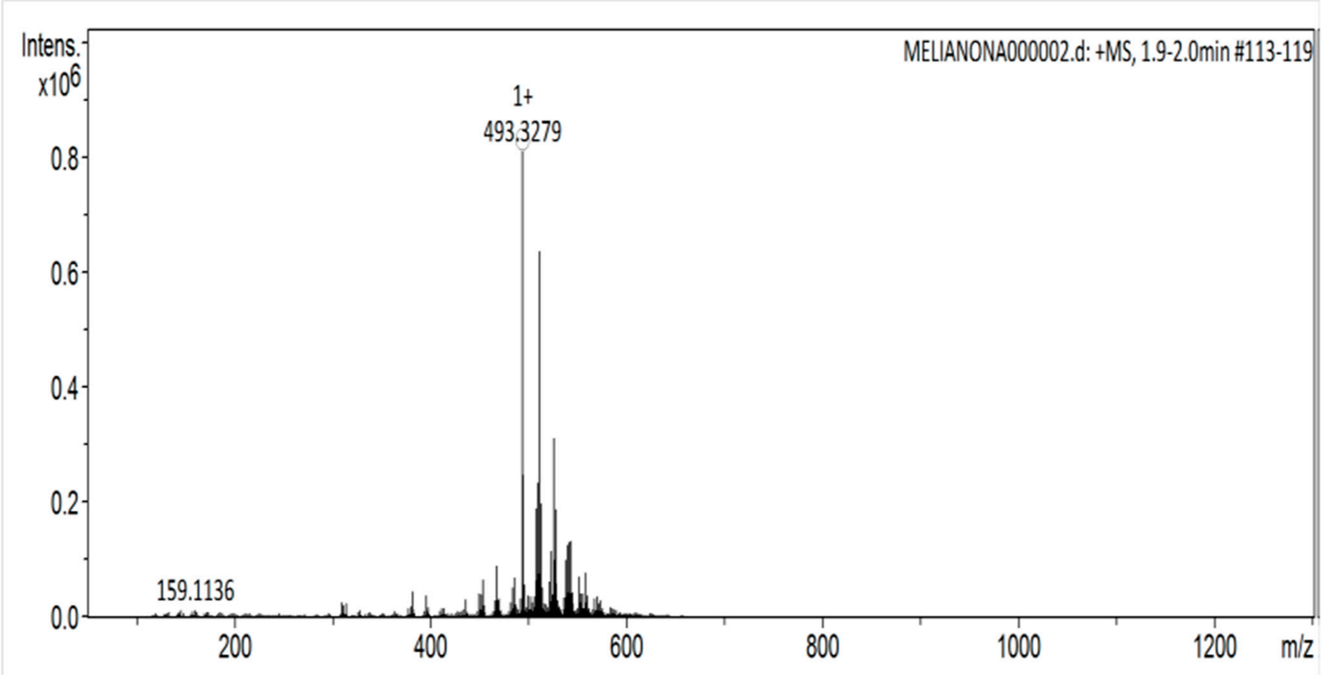

Figure S24. HRESIMS spectrum (positive mode) of **3**.

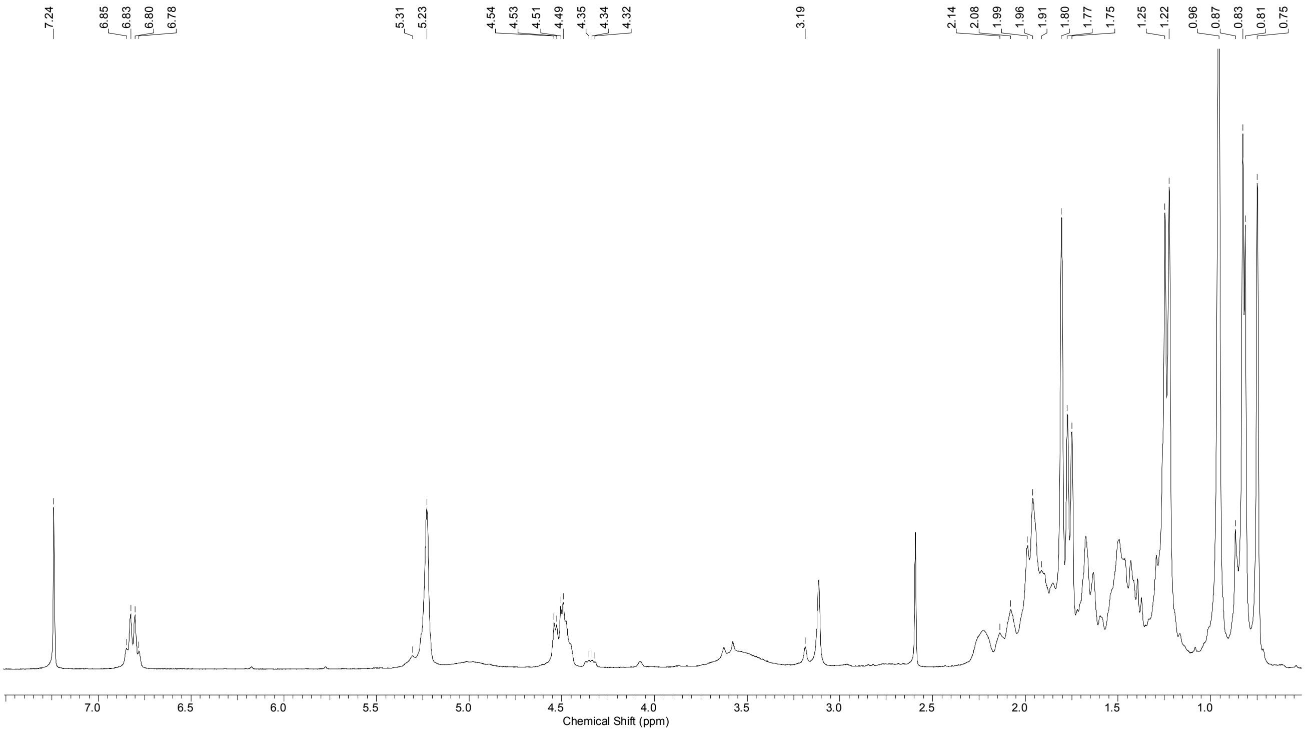

Figure S25. ^{1}H-NMR spectrum (300 MHz, $CDCl_3$) of **4**.

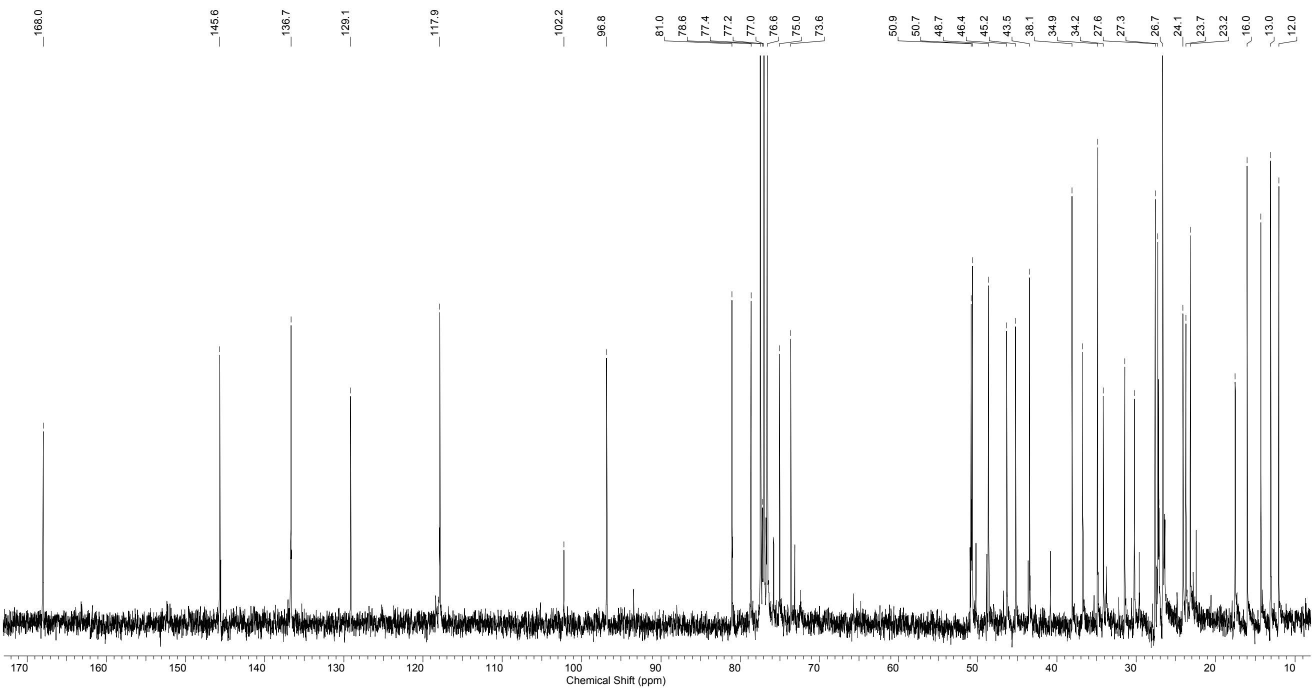

Figure S26. ^{13}C-NMR spectrum (75 MHz, $CDCl_3$) of **4**.

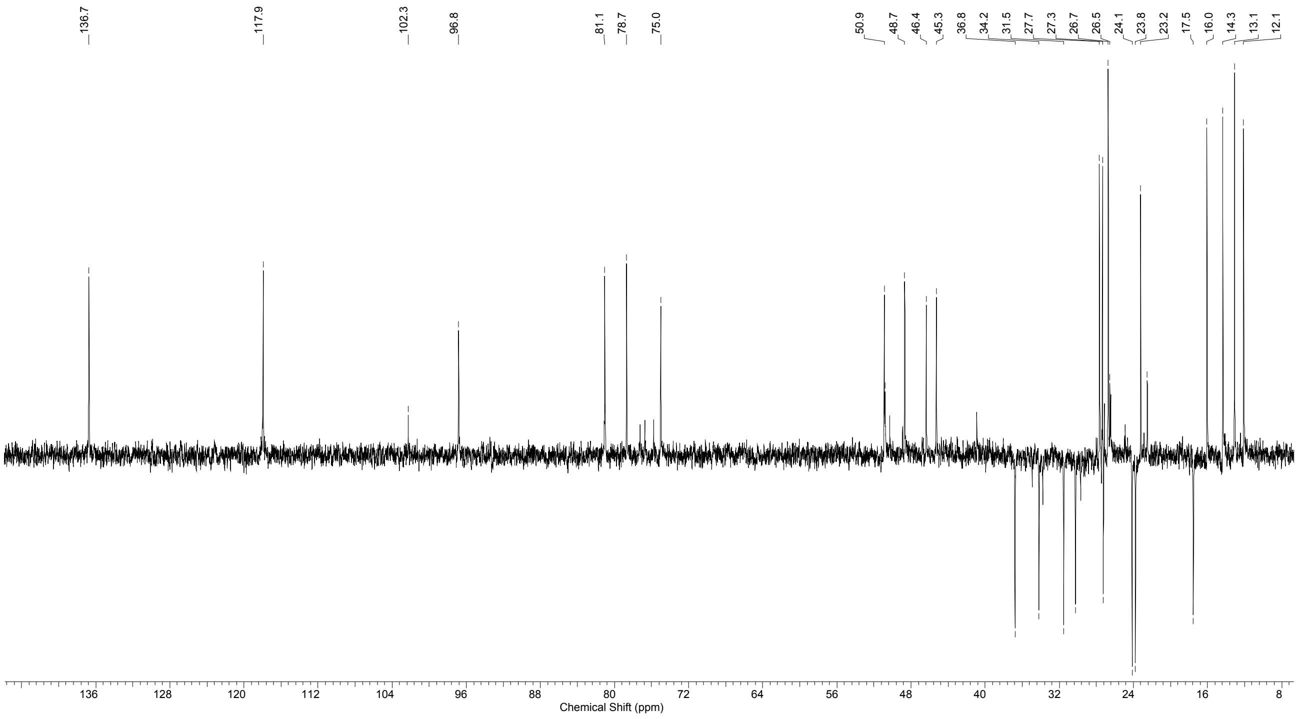

Figure S27. NMR DEPT 135 spectrum (75 MHz, $CDCl_3$) of **4**.

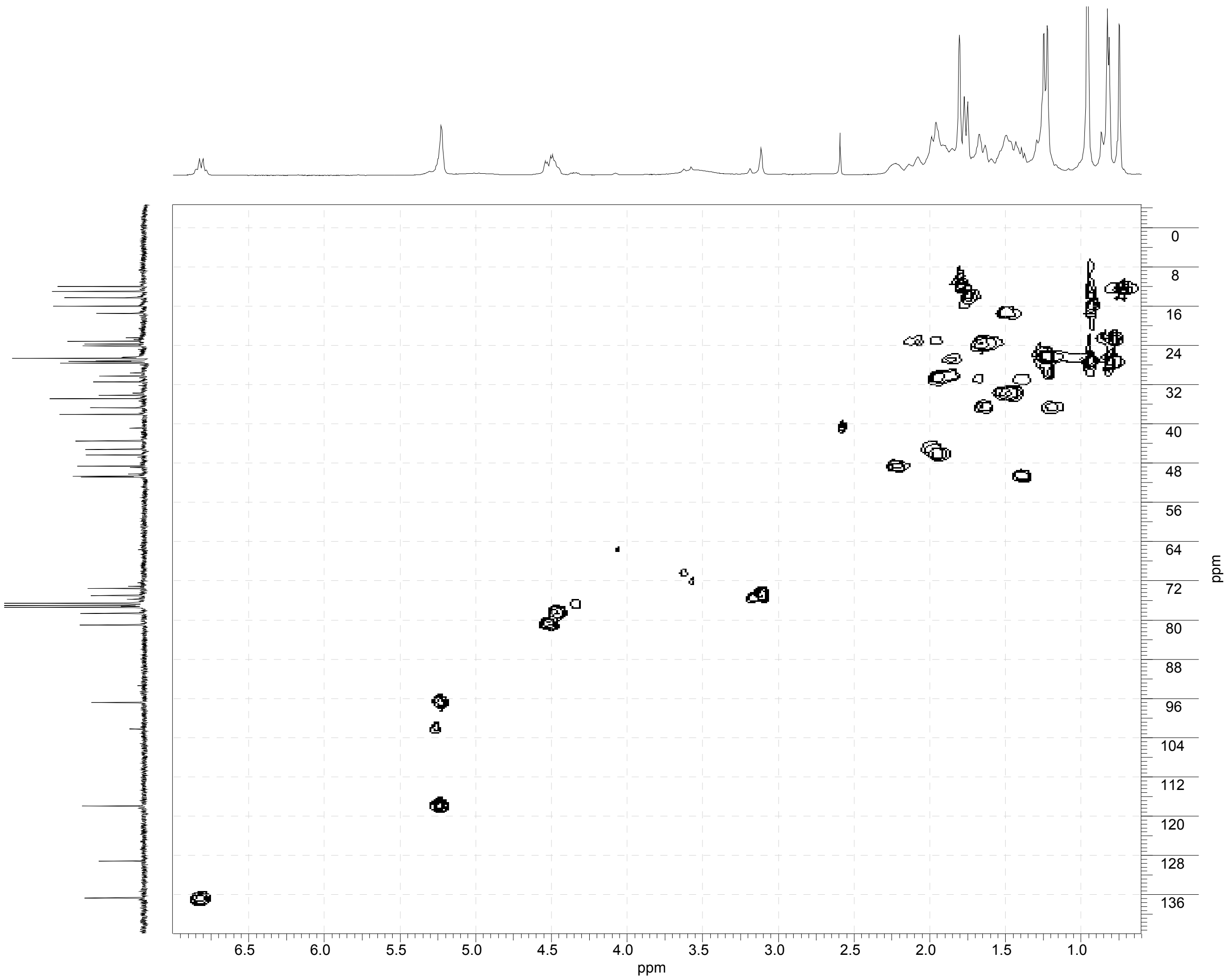

Figure S28. HSQC (300/75 MHz, $CDCl_3$) of **4**.

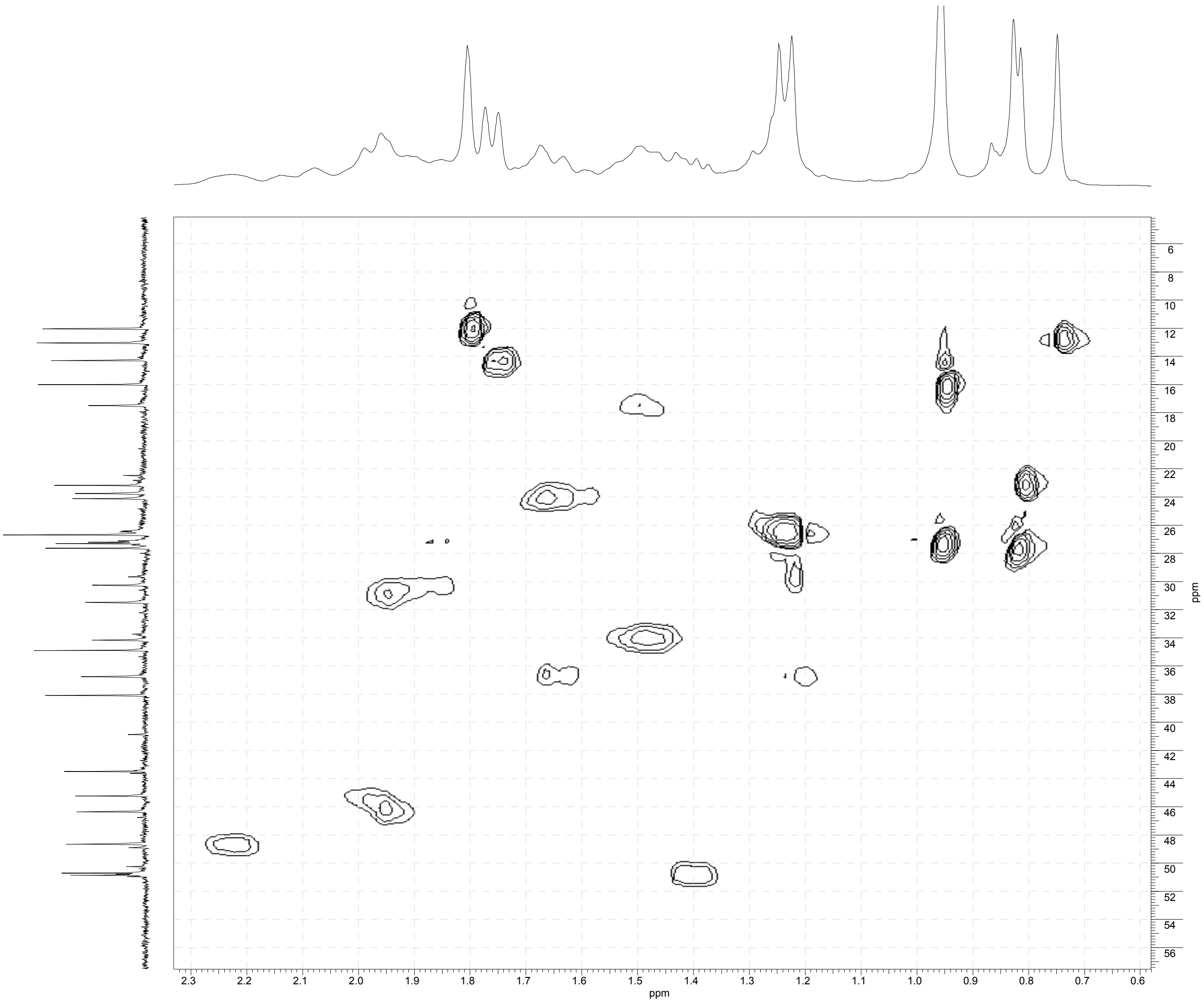

Figure S29. HSQC (300/75 MHz, $CDCl_3$) [Expansion] of **4**.

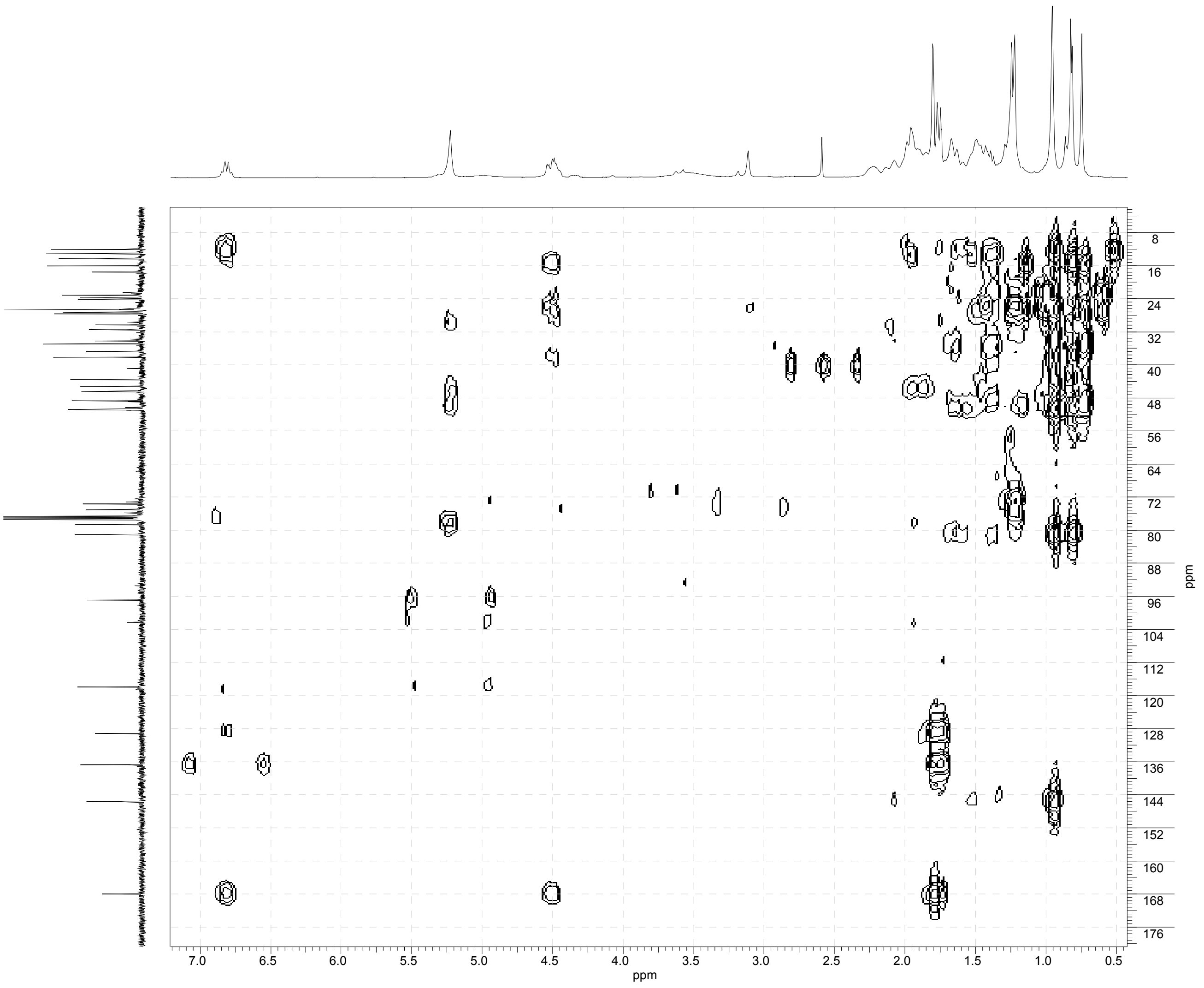

Figure S30. HMBC (300/75 MHz, $CDCl_3$) of **4**.

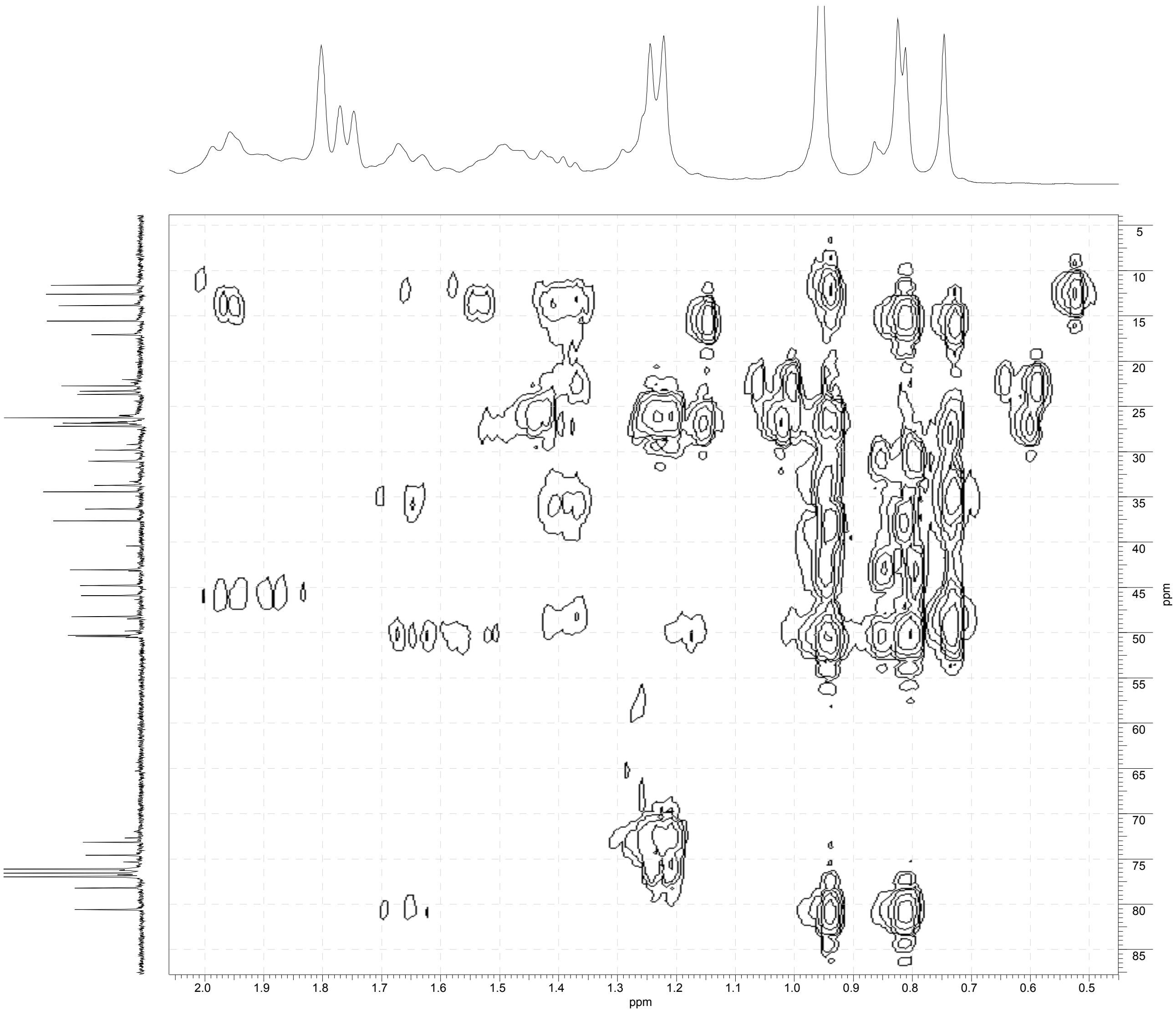

Figure S31. HMBC (300/75 MHz, $CDCl_3$) [Expansion] of **4**.

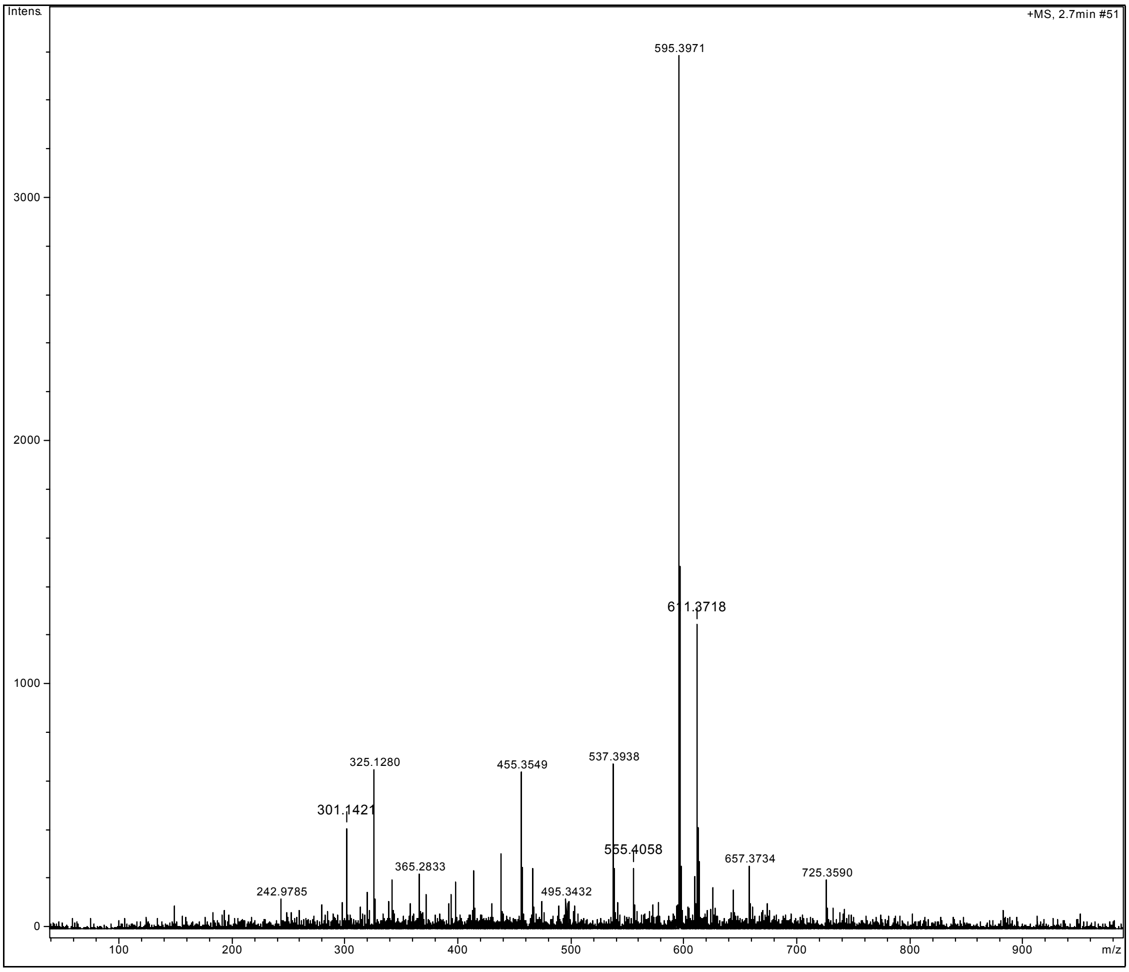

Figure S32. HRESIMS spectrum (positive mode) of **4**.

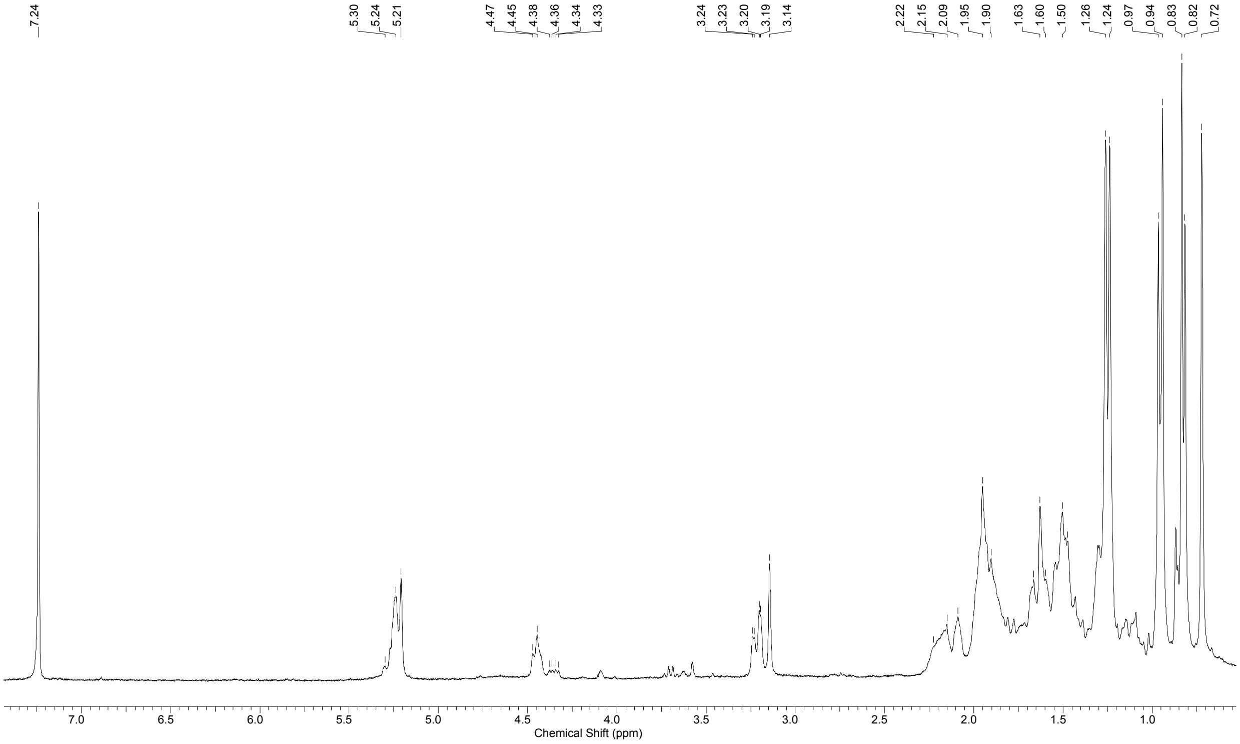

Figure S33. ^{1}H-NMR spectrum (300 MHz, $CDCl_3$) of **5**.

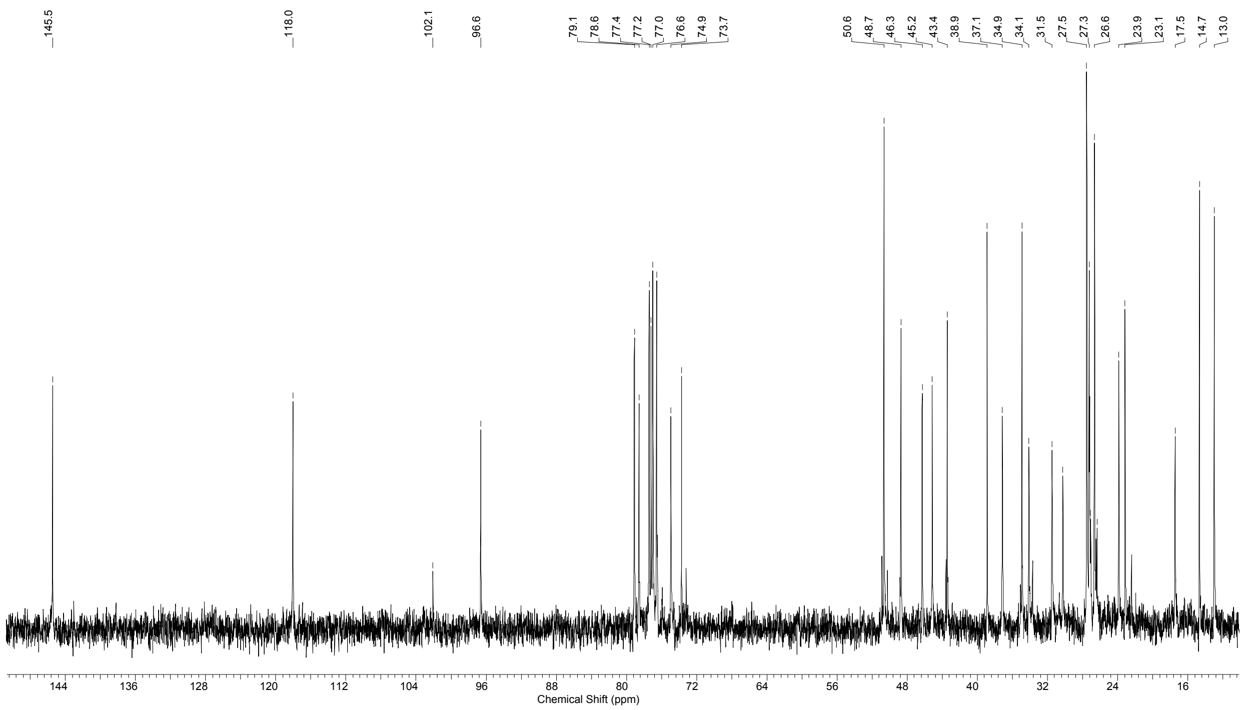

Figure S34. ^{13}C-NMR spectrum of (75 MHz, $CDCl_3$) **5**.

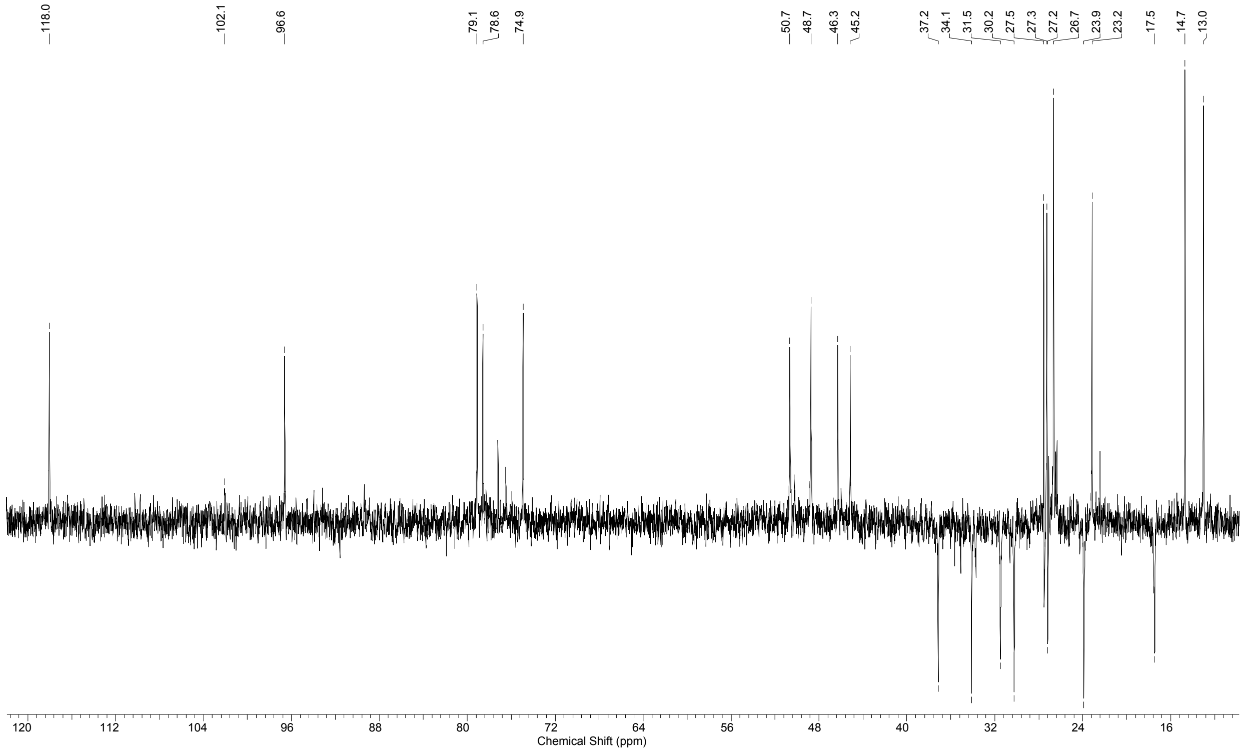

Figure S35. NMR DEPT 135 spectrum (75 MHz, $CDCl_3$) of **5**.

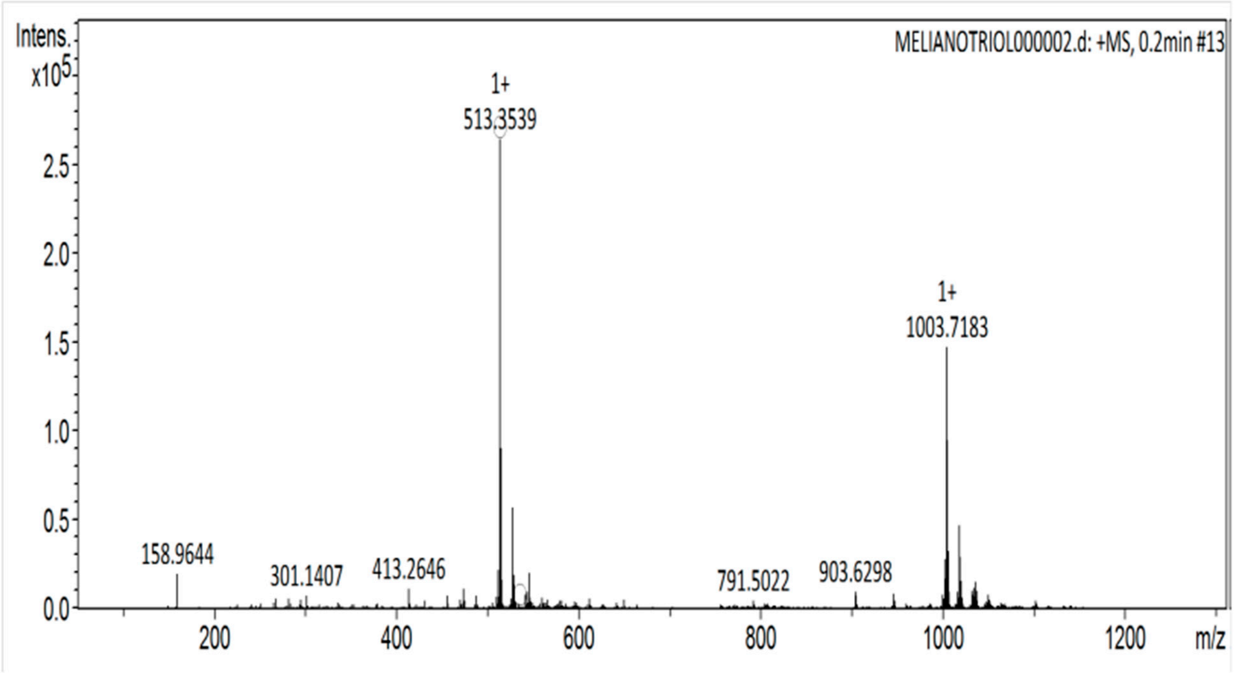

Figure S36. HRESIMS spectrum (positive mode) of **5**.

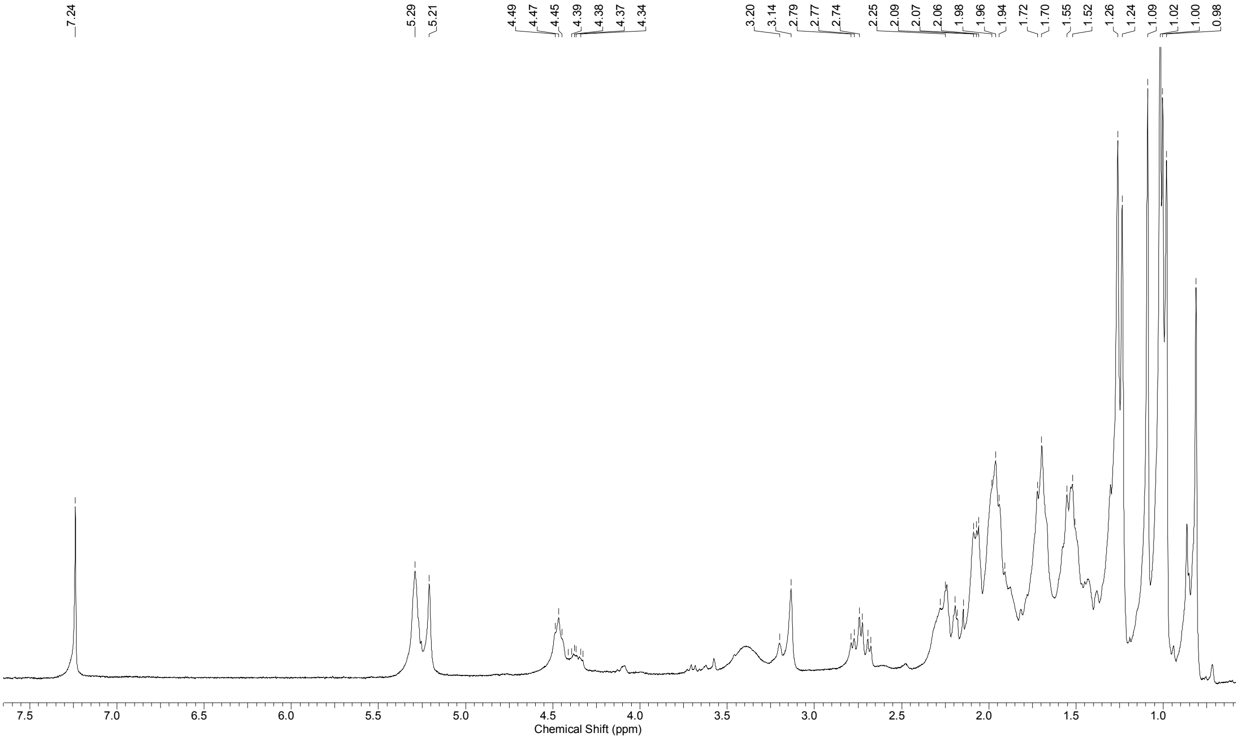

Figure S37. ^{1}H-NMR spectrum (300 MHz, $CDCl_3$) of **6**.

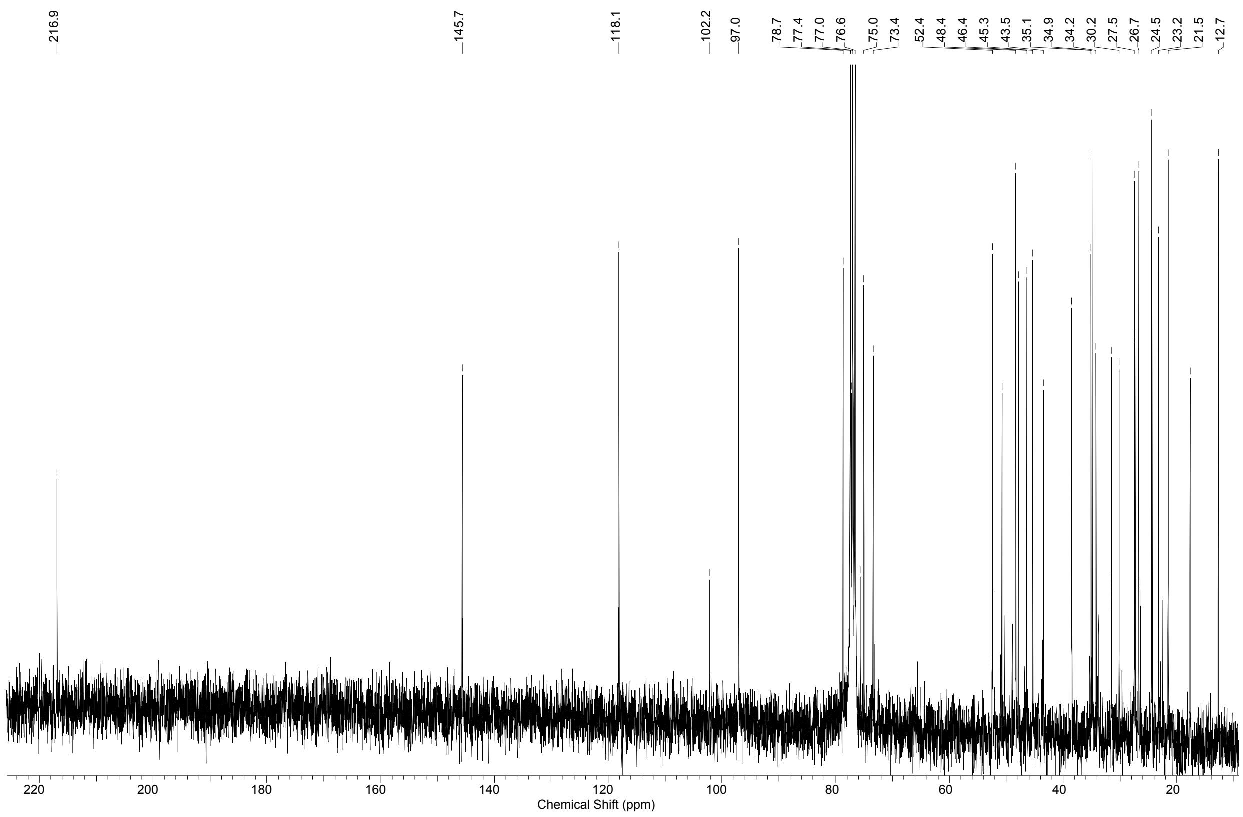

Figure S38. ^{13}C-NMR spectrum of (75 MHz, $CDCl_3$) **6**.

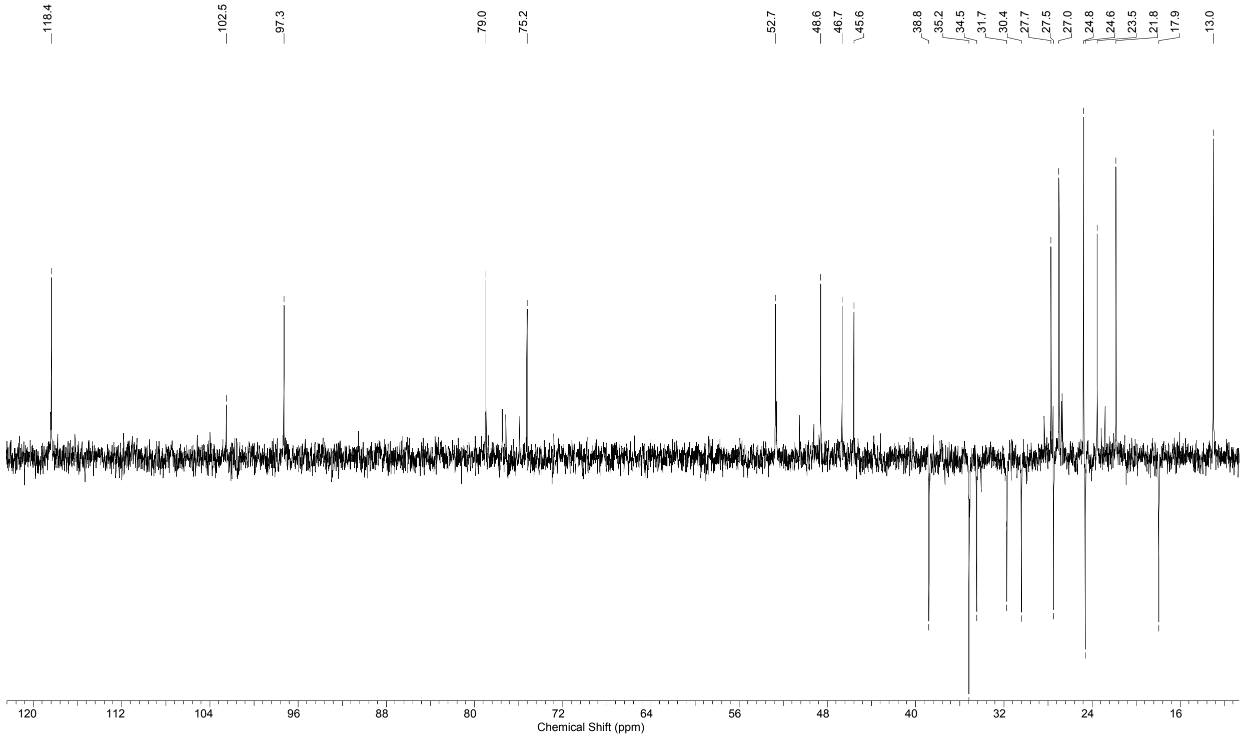

Figure S39. NMR DEPT 135 spectrum (75 MHz, $CDCl_3$) of **6**.

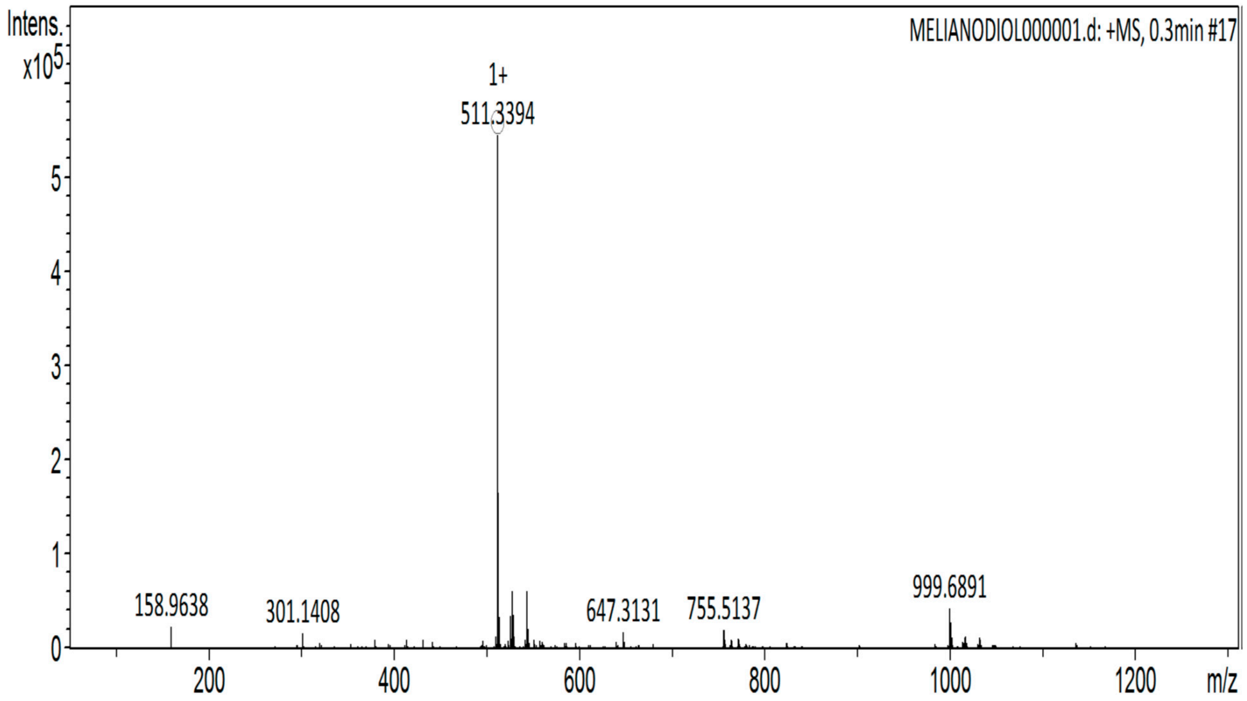

Figure S40. HRESIMS spectrum (positive mode) of **6**.
